# Psychometric evaluation of the body uneasiness test in the Arab Gulf region

**DOI:** 10.3389/fpsyg.2025.1690293

**Published:** 2025-11-14

**Authors:** Bernou Melisse, Carine el Khazen, Edwin de Beurs

**Affiliations:** 1 American Center for Psychiatry and Neurology, Abu Dhabi, United Arab Emirates; 2 Department of Clinical Psychology, Utrecht University, Utrecht, Netherlands; 3 Co-eur, Utrecht, Netherlands; 4 Department of Medical and Clinical Psychology, Tilburg University, Tilburg, Netherlands; 5 Department of Clinical Psychology, Leiden University, Leiden, Netherlands; 6 Stichting Arkin GGZ, Amsterdam, Netherlands

**Keywords:** body uneasiness test (BUT), Arab, validation, body image, psychometric properties, normative data, factor structure

## Abstract

**Introduction:**

The Gulf Cooperation Council is experiencing socio-economic transformations, which coincides with exposure to Western beauty ideals and internalization of the Western thin-ideal, and consequently the development of body uneasiness. The present study psychometrically evaluates the Body Uneasiness Test, suitable for use in the Gulf Cooperation Council.

**Methods:**

After translation and back-translation, the Body Uneasiness Test was administered in a clinical sample (*N* = 169; all completed English version), and a community-based convenience sample (*N* = 544; *n* = 202, 37.1% in Arabic) between July 2024 and July 2025. Criterion validity was determined by two receiver-operating-characteristic curve analyses, one with sample type, and one with the Body Shape Questionnaire as a reference. A confirmatory factor analysis was used to determine the factor structure and *T*-scores and cutoff scores were established for females and males.

**Results:**

Internal consistency was good (*ω* = 0.70–0.96), convergent validity was supported (*r* = 0.66–0.93), and the Body Uneasiness Test discriminated well between both samples (AUC = 0.70–0.94). Sensitivity to change was established in a subsample of 20 patients and was large (Cohen’s *d* = 1.97–2.20). Modified five- and eight-factor structures were confirmed.

**Discussion:**

Though some Gulf countries were underrepresented, the Body Uneasiness Test shows strong promise as a valid assessment tool for use in the Gulf Cooperation Council. However, item redundancy or overlap should be reviewed, and raw scores should first be normalized when utilizing the Body Uneasiness Test. Future research should examine test–retest reliability and further examine sensitivity to change.

## 1 Introduction

Body uneasiness, is associated with constructs like preoccupation with shape/weight and body dissatisfaction (Melisse and Dingemans, 2025). This phenomenon is one of the predisposing factors for eating disorders (Stice et al., 2010). However, body uneasiness is not universal, but a culturally bound syndrome (Awad et al., 2020), and historically associated with Caucasian females (Melisse et al., 2020). Consequently, related research is heavily concentrated on Western samples (Pike and Dunne, 2015).

In Western societies, body uneasiness is associated with the internalization of thin-ideal beauty standards. These standards prioritize slenderness and low body-fat (Stice et al., 2010). In contrast, traditional beauty ideals in Arab societies historically favor a curvier body. Such a physique is often associated with fertility and wealth (Melisse et al., 2020). This suggests that Arab females experience lower levels of body uneasiness (Landor et al., 2024). However, recent literature shows that the Gulf Cooperation Council (GCC; GCCcouncil, 2021) is dealing with socio-economic transformations. This coincides with exposure to Western beauty ideals, and consequent thin-ideal internalization. This may result in an increase of body uneasiness and eating disorders prevalences (Cuzzolaro et al., 2006; Melisse et al., 2024).

Modesty and coverage may offer some protection against body uneasiness by reducing appearance-related pressures and minimizing body surveillance (Swami et al., 2010). Conversely, it is also suggested that modesty and gender norms might paradoxically be associated with body uneasiness in some contexts (Alteneiji, 2023; Al-Mutawa et al., 2019). While body coverage is often viewed as a religious obligation and a cultural marker of social respectability (Alteneiji, 2023), some females report internal conflict. They find it challenging to balance adherence to modest dress codes and the globalized thin-ideal. Media and peer comparison amplify these pressures. This may result in discomfort or compulsive self-monitoring (Barzoki and Alamdar, 2024). However, most self-reports measuring body image fail to fully capture such aspects of body uneasiness.

The two-part Body Uneasiness Test (BUT; Cuzzolaro et al., 2006) is a self-report instrument measuring various aspects of negative body image, such as body avoidance, and compulsive self-monitoring (BUT-A). The second section (BUT-B) assesses concerns toward specific body parts. Prior studies conducted in Western societies show five subscales of the BUT-A, and eight for the BUT-B (Cuzzolaro et al., 2006; Marano et al., 2007; Pokrajac-Bulian et al., 2015). Examining the psychometric characteristics of the BUT in the GCC yields significant benefits for understanding and addressing body uneasiness within the Arab context. Its validation may contribute to more accurate diagnoses, improved assessment of treatment outcomes for eating disorders, and enhance knowledge into the cultural aspects on body uneasiness (Cornelissen and Tovée, 2021).

The aim of the present study is to examine the psychometric properties (internal consistency, convergent validity, incremental validity, criterion-related validity, sensitivity to change and factor structure) and to establish norms, including percentile scores and normalized *T*-scores, of the BUT in the GCC among individuals with an eating disorder and in the general population.

## 2 Materials and methods

### 2.1 Design and procedure

The present study is predominantly a cross-sectional psychometric validation study comparing a clinical sample of individuals diagnosed with an eating disorder characterized by overvaluation of shape/weight (*n* = 169) with a community-based convenience sample (*n* = 544). Additionally, sensitivity to change was assessed using longitudinal data from a smaller clinical subsample (*n* = 20). The BUT was validated against the Body Shape Questionnaire (BSQ) to measure negative body image. Participants received information about the study and provided informed consent by ticking a box in Qualtrics (2024). For minors, consent was obtained from a parent or caretaker. Subsequently, demographics and the self-reports were administered. Participants could complete the self-reports in English or Arabic. All data were collected anonymously through a web-browser or mobile-app, and obtained and stored in online survey platform Qualtrics. The study was approved on June 11, 2024 by the Ethics Review Board of the American Center for Psychiatry and Neurology, Abu Dhabi (ACPN_0064).

### 2.2 Participants and recruitment

Data collection took place between June 2024 and July 2025. Inclusion criteria for both samples were Gulf Cooperation Council (GCC; Bahrain, Kuwait, Oman, Saudi Arabia, Qatar, the United Arab Emirates) residency and aged ≥14. Residents from Bahrain and Oman were eligible but not actively recruited, resulting in possible underrepresentation. The clinical sample included Emiratis and expatriates from the Gulf, North Africa, and the Levant, and was recruited from one of two specialized eating disorder centers in the United Arab Emirates (UAE). Web-based treatment was offered for patients from other GCC countries. Participants first had a diagnostic interview with a clinical psychologist. Subsequently, they were invited to participate in the study, before they commenced treatment. At post-treatment, they completed the BUT again. The community-based sample was recruited via social media platforms, and WhatsApp groups (Melisse et al., 2025), which were chosen because it is challenging to gather data from GCC residents, who are less inclined to participate in research (Melisse et al., 2022a).

### 2.3 Measures

*Background* var*iables:* A pre-established checklist was used to administer demographics and to assess the presence of comorbid psychopathology: participants were asked to indicate (tick) which of the listed diagnoses were present.

### 2.4 Body uneasiness test (BUT)

The BUT (Cuzzolaro et al., 2006) is a self-report measuring several dimensions of negative body image among individuals with and without an eating disorder. It has two sections, the BUT-A and the BUT-B. The items on both sections are answered on a 6-point Likert scale from 0 (never) to 5 (always) regarding the frequency of these feelings. Higher scores indicate greater body uneasiness. The BUT-A has 34 items, measuring negative body image, compulsive self-monitoring, avoidance, and estrangement feelings toward the body. The BUT-A Global Severity Index (GSI) is the averaged score on all items, and ranges between 0 and 5. Though cutoff scores vary over cultures, the Italian and the Dutch version have a cutoff of >1.2 indicating clinical levels of body uneasiness (Cuzzolaro et al., 2006; Van Uffelen et al., 2025). Subscales of the BUT-A are weight phobia (items 9, 10, 18, 21, 24, 31, 32, 33), body image concerns (3, 4, 6, 12, 15, 22, 23, 25, 34), avoidance (5, 8, 13, 16, 19, 30), compulsive self-monitoring (1, 11, 17, 20, 27) and depersonalization (2, 7, 14, 26, 28, 29). The BUT-B has 37 items, assessing uneasiness, shame, or disgust (“I hate…”) with specific body parts and functions (odor, noises, sweat, blushing). The total score of the BUT-B involves two parts, (i) the Positive Symptom Total (PST), operationalized as the sum of items scored higher than 0 (range 0–37), and (ii) the Positive Symptom Distress Index (PSDI): the averaged total score on the PST items (range 0–5). Dutch cutoffs are >15.5 and >1.9 for the BUT-B PST and PSDI, respectively (Van Uffelen et al., 2025). The BUT-B subscales are indicated by roman numerals: B I: Mouth (11, 10, 8, 9, 12, 7), B II: Face Shape (3, 2, 15, 14, 6, 13), B III: Thighs (29, 28, 25, 24, 30), B IV: Legs (31, 32, 33, 21, 1), B V: Arms (20, 23, 22, 19, 26), B VI: Mustache (18, 16, 17), B VII: Skin (4, 5), B VIII: Blushing (27, 34, 35, 36, 37). Prior studies showed that the BUT subscales have sufficient internal consistency (Cronbach’s *α* > 0.70), and good test–retest reliability (*r* > 0.70) (Cuzzolaro et al., 2006; Marano et al., 2007; Pokrajac-Bulian et al., 2015).

To establish an Arabic version (see Supplementary Appendix 1), two independent translators (one with eating disorder expertise, one certified) translated the BUT. A third expert compared translations, and a consensus meeting was held to finalize this version. For the back-translation, three new translators repeated this process. Discrepancies between the back-translation and the original were resolved by consensus (Swami and Barron, 2019). The translations were piloted: students rated comprehension of all Arabic items on a 4-point Likert scale (1, I do not understand at all, 4, Completely clear). The translation was understandable (comprehensiveness rating: *M* = 3.2, *SD* = 0.3). However, none of the participants in the clinical sample preferred the Arabic version over the English one. In the community-based sample, *n* = 342/544, 62.9% participants completed the BUT in English, and *n* = 202/544, 37.1% in Arabic.

### 2.5 Body shape questionnaire (BSQ)

The BSQ (Cooper et al., 1987), a self-report questionnaire measuring negative body image over the past 28 days, was validated for use in Arab societies (Melisse et al., 2022b). Internal consistency of the BSQ was excellent in the present study (McDonald’s *ω* = 0.95).

### 2.6 Eating disorder examination questionnaire (EDE-Q)

The EDE-Q (Fairburn and Beglin, 2008), a self-report questionnaire measuring eating disorder pathology over the past 28 days, was validated for use in Arab societies (Melisse et al., 2021). Internal consistency of the EDE-Q was excellent in the present study (McDonald’s *ω* = 0.89).

### 2.7 Power

The confirmatory factor analysis (CFA) required 3–20 times the number of items as a minimum sample size with an absolute range of 100–1,000 (Mundfrom et al., 2005). The minimum thresholds for the present study were met with at least 102 participants for the BUT-A and 111 for the BUT-B. Sample size recommendations for establishing normalized *T*-scores generally range from 100 to 200 per subgroup (Wolf et al., 2013). Consequently, age-based subgroups were adequately sized (>200). However, the male subgroup (*N* = 86) was below this threshold, which may reduce the appropriateness of male-specific norms.

### 2.8 Statistical analysis

*Software and assumptions:* The analyses were performed in SPSS v29 (IBM Corp, 2024), *R* and *R* package Lavaan, version 0.6–5 (Rosseel, 2012) for CFA. Norms were established with the RNOmni package version 1.0.1 (McCaw, 2019), nls and nlstools (Wu and Estabrook, 2016), and mirt (Chalmers, 2012). Assumptions were checked. All results were reported in accordance with the Standards for Reporting of Diagnostic Accuracy Studies guidelines (Cohen et al., 2016). Group comparisons were made with independent sample *t*-tests, Chi-square tests, and Mann–Whitney *U* tests.

*Reliability and validity:* Internal consistency of the measures were calculated by Cronbach’s *α* and McDonald’s *ω* (≥0.70 acceptable, ≥0.90 excellent) (MacDonald, 1999). Convergent validity was determined by the association of the BUT-A-GSI/BUT-B and BSQ scores. Criterion-related validity (known-groups validity) was determined by comparing BUT-A/BUT-B means of the clinical versus population-based samples using *t*-tests, or Wilcoxon rank-sum tests for non-normal data (Wilcoxon et al., 1963). Adjusted *t*-values and degrees of freedom were used if homogeneity was violated (Ramseyer and Tcheng, 1973). Receiver-operating-characteristic (ROC) analysis and area-under-the-curve (AUC). AUC calculation examined how well the BUT distinguished clinical status (per BSQ) and groups membership. AUCs of >0.70 were deemed acceptable (Swets, 1988). Sensitivity to change was established in a clinical subsample with a dependent sample *t*-test, the correlation between change scores (change from pre- to post-treatment) on the BUT and BSQ were calculated.

*Norms:* Cut-off values for clinical significance were determined as explained in the Supplementary Appendix 2 (Jacobson and Truax, 1991; Jacobson et al., 1999). Additionally, norms were established by calculating normalized *T*-scores, and Percentile Rankorder (PR) scores. First, the effect of age and sex was examined to assess the need of age- and gender specific norms. Cross-walk tables and formulas to transform raw scores into normalized *T*-scores and PR-scores were established based on the frequency of responses in the clinical sample, using: $\mathrm{PR}=(\frac{m+0.5k}{N})*100$ , where *m* indicates the number of participants with a score < Raw Score (RS), and *k* indicates the number of participants with exactly RS and *N* indicates the size of the normative sample (Crawford and Garthwaite, 2009). First, as raw scores of the community-based sample were skewed to the right, scores were normalized through the Rankit approach (Ipsen and Jerne, 1944; Bliss et al., 1956). RankNorm yields percentile ranks, which were converted to normalized Z-scores using the probit function, which calculates the inverse of the cumulative distribution function (Solomon and Sawilowsky, 2009). Resulting Z-scores were converted to *T*-scores by *T* = 10*Z + 50. The procedure is described elsewhere in more detail (de Beurs et al., 2025).

*Construct validity:* The factor structures of the BUT-A and BUT-B were examined by confirmatory factor analysis (CFA). The response options were considered ordered, and polychoric correlations were used. The DWLS estimator was used with NLMINB as the optimization method, and we report robust fit indices. When the RMSEA was <0.05 (0.5–0.8 acceptable) and the TLI and CFI were >0.95, the model had a good fit (Hu and Hu and Bentler, 1999).

## 3 Results

### 3.1 Participants

For the clinical sample, 169 participants were recruited, who all met the inclusion criteria. There were no missing data. The sample was predominantly female (*n* = 151/169, 89.3%), Emirati (*n* = 64/169, 37.9%), diagnosed with anorexia nervosa (*n* = 84/169, 49.7%) and mean age and BMI were 26.0 (*SD* = 9.1) years, and 25.1 (*SD* = 8.8) kg/m^2^, respectively. Mean scores were: BUT-A-GSI = 2.48 (*SD* = 1.25); BUT-B-PST = 19.18 (*SD* = 10.46); BUT-B-PSDI = 1.45 (*SD* = 1.05). For the community-based convenience sample, 89.8%, *N* = 544/606 participants had <5% missing data, *n* = 471/544 (87.4%) were female, and Kuwaiti 227/544 (41.7%). Mean age and BMI were 26.7 (*SD* = 12.1) years, and 23.5 (*SD* = 6.0) kg/m^2^. Mean BUT scores were: BUT-A-GSI = 1.15 (*SD* = 1.08); BUT-B-PST = 11.74 (*SD* = 6.89); BUT-B-PSDI = 0.79 (*SD* = 0.88). The gender imbalance potentially limited generalizability of the results to males. Table 1 displays the demographics of both samples and shows that the clinical sample had higher scores on the self-reports, had more comorbid psychopathology, were Emirati and from the Eastern Mediterranean region and there were differences in their daily life and highest level of education.

**Table 1:** Demographic characteristics and statistical comparisons of the clinical sample diagnosed with an eating disorder (*N* = 169) and a community-based convenience sample (*N* = 544).

		Community-based sample (*N* = 544)	Clinical sample (*N* = 169)	*p*
Age, mean (*SD*)		26.7 (12.1)	26.0 (9.1)	0.279
Sex, *n* (%)	Female	471 (87.4%)	151 (89.3%)	0.172
	Male	68 (12.6%)	18 (10.3%)	
Nationality, *n* (%)^1^	United Arab Emirates	42 (7.7%)	64 (37.9%)	<0.001
	Saudi Arabia	121 (22.2%)	0 (0%)	
	Kuwait	227 (41.7%)	0 (0%)	
	Qatar	2 (0.4%)	0 (0%)	
	African region	9 (1.7%)	3 (1.8%)	
	Americas	2 (0.4%)	8 (4.7%)	
	European	24 (4.4%)	36 (21.3%)	
	Eastern Mediterranean	85 (15.7%)	48 (28.4%)	
	Pacific	6 (1.1%)	5 (3.6%)	
	Northern America	16 (2.9%)	8 (4.7%)	
	South East Asia	10 (1.8%)	0 (0%)	
Body Mass Index, Mean (*SD*)		23.5 (6.0)	25.1 (8.8)	<0.001
Eating disorder diagnosis	Anorexia nervosa	*NA*	84 (49.7%)	
	Bulimia nervosa	*NA*	30 (17.8%)	
	Binge-eating disorder	*NA*	55 (32.5)	
Comorbid psychopathology, *n* (%)	Absent	294 (54.1%)	13 (7.7%)	<0.001
	Anxiety disorder	110 (20.3%)	19 (11.2%)	
	ADHD	39 (7.2%)	71 (42.0%)	
	Autism	7 (1.3%)	9 (5.3%)	
	Mood disorder	90 (16.6%)	50 (29.6%)	
	OCD	23 (4.2%)	14 (8.3%)	
	Personality disorder	11 (2.0%)	8 (4.7%)	
	PTSD	22 (4.1%)	12 (7.1%)	
Marital status, *n* (%)	Married	101 (18.6%)	35 (20.7%)	0.176
	Single	410 (75.5%)	131 (77.5%)	
	Divorced	14 (2.6%)	2 (1.2%)	
	Engaged	18 (3.3%)	1 (0.6%)	
Daily role, *n* (%)	High school	80 (14.7%)	42 (24.9%)	<0.001
	University	262 (48.3%)	18 (10.7%)	
	Vocational education	17 (3.1%)	14 (18.3)	
	Employed	148 (27.3%)	58 (34.3%)	
	Unemployed	30 (5.5%)	0 (0%)	
	Other	30 (5.5%)	37 (21.9%)	
Highest level of education, *n* (%)	Kindergarden	3 (0.6%)	0 (0%)	<0.001
	Primary school	16 (2.9%)	11 (6.5%)	
	High school	202 (37.2%)	63 (37.3%)	
	Vocational education	34 (6.3%)	4 (2.4%)	
	Bachelor	239 (44.0%)	66 (39.1%)	
	Master/ doctorate	49 (9.0%)	25 (14.7%)	
Body shape questionnaire, mean (*SD*)		65.9 (40.3)	118.2 (43.1)	<0.001
Eating disorder examination questionnaire, Mean (*SD*)		2.5 (1.7)	4.5 (1.5)	<0.001

SD = standard deviation. ^1^ Regions in accordance with the World Health Organization (n.d.).

### 3.2 Psychometric properties

Table 2 shows the mean scores on the BUT-A and B and their subscales for the samples combined, Table 3 for both samples separately. Given the difference between females and males and between in age, Supplementary Table 2a presents mean scale scores for females and males separately and Supplementary Table 2b present the mean scores for two age groups.

**Table 2:** Scale descriptives and reliabilities.

BUT-A	*M*	*SD*	Skew	Kurt	*ω -h*	*α*	ω *-tot*	Mean *r*	*N* items
General severity index	1.47	1.26	0.65	−0.64	0.86	0.98	0.98	0.57	34
Weight phobia	1.72	1.44	0.51	−0.90	0.85	0.92	0.93	0.62	8
Body image concern	1.60	1.43	0.70	−0.62	0.87	0.94	0.95	0.66	9
Avoidance	1.11	1.19	0.97	−0.05	0.74	0.88	0.90	0.53	6
Compulsive self-monitoring	1.52	1.29	0.70	−0.41	0.76	0.86	0.88	0.56	5
Depersonalization	1.22	1.29	0.96	−0.06	0.80	0.90	0.92	0.60	6

BUT-B	*M*	*SD*	Skew	Kurt	ω *-h*	*α*	ω *-tot*	Mean *r*	*N* items
Positive symptoms total	13.89	11.82	0.45	−1.14	*	*	*	*	37
Positive symptom distress index	0.95	0.96	1.09	0.53	0.78	0.96	0.97	0.42	37
B1 Mouth	0.85	1.02	1.35	1.30	0.74	0.87	0.89	0.51	6
B2 Face shape	0.72	0.95	1.48	1.68	0.69	0.84	0.87	0.47	6
B3 Thighs	1.46	1.47	0.69	−0.71	0.80	0.89	0.91	0.60	5
B4 Legs	0.86	1.08	1.41	1.39	0.69	0.82	0.86	0.50	5
B5 Arms	0.99	1.26	1.36	1.07	0.77	0.88	0.90	0.57	5
B6 Mustache	0.69	1.07	2.01	3.92	0.02	0.74	0.77	0.46	3
B7 Skin	1.24	1.33	0.95	0.06	**	0.70	0.70	0.54	2
B8 Blushing	0.92	1.05	1.20	0.92	0.65	0.78	0.82	0.39	5

BUT = Body Uneasiness Test, α = Cronbach’s alfa, ω -h = McDonald’s omega-h (hierarchical estimate of the general factor saturation of a scale), α = Cronbach’s alpha coefficient of internal consistency; ω -tot = omega-total (a model based estimate of the total reliability of a scale); Mean *r* = mean correlation among items; *N* items = number of items of a scale. *Count of items scored >0, no reliability established; **only two items, no w-h computed.

**Table 3:** Means and SD for clinical and community-based respondents.

Scale	Clinical		Population		*t*	*p*	*d*	95%CI
BUT-A	*M*	*SD*	*M*	*SD*				
General severity index	2.48	1.25	1.15	1.08	12.44	<0.0001	1.18	[1.00, 1.36]
Weight phobia	2.94	1.33	1.34	1.25	14.38	<0.0001	1.27	[1.08, 1.45]
Body image concern	2.68	1.46	1.26	1.24	11.41	<0.0001	1.09	[0.91, 1.28]
Avoidance	1.95	1.30	0.84	1.03	10.13	<0.0001	1.01	[0.83, 1.19]
Compulsive self-monitoring	2.41	1.39	1.25	1.12	9.82	<0.0001	0.97	[0.79, 1.15]
Depersonalization	2.20	1.36	0.91	1.10	11.24	<0.0001	1.11	[0.92, 1.29]

BUT-B	*M*	*SD*	*M*	*SD*	*t*	*p*	*d*	95%CI
Positive symptoms total	19.18	10.46	12.22	11.74	6.87	0.0000	0.61	[0.43, 0.78]
Positive symptom distress index	1.45	1.05	0.79	0.88	7.43	0.0000	0.72	[0.54, 0.90]
B1 mouth	1.13	1.13	0.76	0.97	3.78	<0.0001	0.36	[0.19, 0.54]
B2 Face shape	1.05	1.10	0.62	0.88	4.62	<0.0001	0.46	[0.28, 0.63]
B3 Thighs	2.61	1.43	1.10	1.29	12.22	<0.0001	1.14	[0.95, 1.32]
B4 Legs	1.40	1.24	0.69	0.96	6.83	<0.0001	0.69	[0.51, 0.87]
B5 Arms	1.75	1.45	0.75	1.09	8.27	<0.0001	0.85	[0.67, 1.03]
B6 Mustache	0.84	1.14	0.64	1.05	2.07	0.0196	0.18	[0.01, 0.36]
B7 Skin	1.58	1.39	1.13	1.30	3.91	0.0001	0.35	[0.17, 0.52]
B8 Blushing	1.25	1.12	0.82	1.00	4.71	<0.0001	0.42	[0.24, 0.59]

On all scales a significant difference was found with higher scores for clinical subjects as compared to respondents from the general population. Differences on the BUT-A scale were larger than on the BUT-B scales.

#### 3.2.1 Internal consistency

The descriptive item and scale statistics are depicted in Supplementary Tables 1, 2. Most items had a skewed responses distribution. The overrepresentation of zero-scores indicated floor effects. This skewness potentially affects factor structures and limits the interpretability of psychometric outcomes. Table 2 shows that internal consistencies of the BUT-A-GSI and scale scores were high (McDonalds *ω-*total = 0.88–0.98) and varied between acceptable to excellent in the BUT-B (McDonalds *ω-*total = 0.70 -. 96).

#### 3.2.2 Convergent and incremental validity

The convergent validity was supported (*p* < 0.001): BSQ and BUT-A-GSI, and the BSQ and BUT-B-PST scores were strongly correlated in the clinical sample (BUT-A-GSI: *r* = 0.93; BUT-B-PST: *r* = 0.72), and in the community-based sample (BUT-A: *r* = 0.86; BUT-B-PST: *r* = 0.72).

In the clinical sample, 78.6% of the BUT-A-GSI scores was explained by the EDE-Q global-score [*R*^2^ = 0.79, *F*(1, 169) = 407.3, *p* < 0.001]. The BUT-A scores were strongly correlated with the EDE-Q global-score (*r* = 0.89, *p* < 0.001). The BUT-B-PST scores were also fairly associated with the EDE-Q global-score, BUT-B-PST: Adjusted *R*^2^ = 0.44, *r* = 0.66, *p* = <0.001, *F*(1, 169) = 87.2. This indicated that the BUT added value by assessing specific aspects of body uneasiness not captured by the EDE-Q. The results were somewhat less convincing in the community-based sample, but still substantial correlations were found, BUT-A-GSI: Adjusted *R*^2^ = 0.47, *r* = 0.66, *p* = <0.001, *F*(1,543) = 261.5; BUT-B-PST: Adjusted *R*^2^ = 0.34, *r* = 0.58, *p* = <0.001, *F*(1,543) = 98.4. Though the convergent and incremental validity were high, they could indicate redundancy between measures.

Table 3 shows the results of comparing scale scores of both samples with independent *t*-tests. Cohen’s *d* indicated a large effect size (*d* ≈ 0.80) for the majority of scales, with the exception of B1, B2, and B6–B8, which showed medium effects (*d* ≈ 0.50).

#### 3.2.3 Criterion-related validity

The BUT-A-GSI and the BUT-B-PST accurately measured a negative body image according to the BSQ among individuals with an eating disorder. High AUCs were revealed (*p* < 0.001) for the BUT-A-GSI [AUC = 0.94 (0.93–0.95),] and the BUT-B-PST [AUC = 0.89 (0.87–0.91)]. In addition, when sample type was used as a criterion, a high AUC (*p* < 0.001) was revealed for the BUT-A-GSI [AUC = 0.87 (0.83–0.90)], and an acceptable AUC for the BUT-B-PST [AUC = 0.70 (0.65–0.76)]. This indicated that the BUT accurately discriminated between the clinical and the community-based sample. An individual in the clinical sample had an 87% more likelihood of having a higher score on the BUT-A, and 70% more on the BUT-B compared to an individual from the community-based sample. Additionally, Table 3 shows that according to one-sided *t*-tests that the clinical sample had higher scores compared to the non-clinical sample on the BUT-A-GSI, BUT-B-PST, and the BUT-B-PDSI.

#### 3.2.4 Sensitivity to change

In the clinical sample, sensitivity to change was demonstrated [paired samples *t*-test: BUT-A-GSI: *t*(19) = 12.5, *p* = <0.001, Cohen’s *d* = 1.97 (1.75, 2.20); BUT-B-PST: *t*(19) = 12.3, *p* = <0.001, Cohen’s *d* = 2.20 (1.97, 2.44)], which was similar to the sensitivity to change of the BSQ [paired samples *t*-test: BSQ: *t*(19) = 12.8, *p* = <0.001]. Additionally, a strong positive correlation was found between change scores on the BUT and the BSQ [*r* = 0.82, *p* < 0.001, Cohen’s *d* = 1.39 (1.09, 1.67)], further demonstrating similarity in sensitivity to change of both measures. However, it should be noted that these analyses were performed in a small subset of 20 clinical participants pre-post treatment, and these results should be interpreted with caution.

#### 3.2.5 Confirmatory factor analysis

Table 4 shows sufficient fit for the modified five-factor model of the BUT-A, with better fit than competing models. Modification indices suggested that some items overlapped conceptually, e.g., feeling detached from your body and feeling that your body does not belong to you (items 28 and 29), and being in front of the mirror and looking at oneself (items 1 and 11). Allowing these similar items’ error to correlate improved model-fit. Additionally, cross-loadings were allowed for item 2 on both the compulsive self-monitoring and weight phobia factors, and for item 29 on the weight phobia factor. Table 4 also indicated that for the BUT-B a modified eight-factor structure showed good fit. Modification indices allowing for correlated error terms between item pairs: 16–17 (mustache-beard), 24–25 (stomach-abdomen), 29–31 (thighs-legs), 27–28 (buttocks-hips), and 22–23 (chest-breasts) improved fit further. The overlap between items indicated that some items load on more than one latent trait and therefore items address closely related topics. The shared variance was not fully accounted for by the broader factor structure.

**Table 4:** Indicators of unidimensionality (robust; response categories considered to be ordered); baseline χ^2^ = 71924.50 for the BUT-A, and indicators of unidimensionality (robust; response categories considered to be ordered); baseline χ^2^ = 239077.87 for the BUT-B.

Model	*χ^2^*	*df*	*χ^2^/df*	*χ^2^ diff*	*CFI*	*TLI*	*SRMR*	*RMSEA*	CI90
BUT-A-single factor	3219.85*	527	6.11	68,705	0.962	0.960	0.047	0.085	0.082–0.088
BUT-A-5-factor	2522.59*	517	4.88	6,940	0.972	0.970	0.041	0.074	0.071–0.077
BUT-A-5-factor (modified)^1^	2112.51*	512	4.13	69,812	0.978	0.975	0.037	0.066	0.063–0.069
BUT-A-5-bi-factot	2248.40*	493	4.56	69,576	0.975	0.972	0.039	0.071	0.068–0.074
BUT-A-5-secorderfactor	2681.28*	522	5.14	69,143	0.970	0.967	0.043	0.076	0.073–0.079

Model	*χ^2^*	df	*χ^2^*/df	*χ^2^* diff	CFI	TLI	SRMR	RMSEA	CI90
BUT-B-single factor	3497.64*	629	5.56	235,580	0.988	0.987	0.070	0.081	0.078–0.084
BUT-B-8-factor	1983.13*	601	3.30	237,095	0.994	0.994	0.056	0.057	0.055–0.060
BUT-B-8factor (modified)^2^	1536.39*	596	2.58	237,541	0.996	0.996	0.051	0.048	0.045–0.051

*p < 0.001; ^1^Modification indices: item28 ~ ~ item29; item1 ~ ~ item11; CSM = ~ item2; WP = ~ item2; WP = ~ item29; ^2^Modification indices: item16 ~ ~ item17, item24 ~ ~ item25, item29 ~ ~ item31, item27 ~ ~ item28, item22 ~ ~ item23.

#### 3.2.6 Norms

Supplementary Table 3 offers a crosswalk from a selection of raw scores for the General Severity Index score of the BUT-A to population-based *T*-scores and population based and clinical PR-scores. Additionally, Supplementary Table 3 provides a clinical utilization example. Generally, *T-* and *PR*-scores are interpreted as shown in Supplementary Appendix 2. Supplementary Tables 3a–8a present crosswalk tables for the BUT-A, and its subscales for females and males, separately. Given the gender-differences (BUT-A: *p* = 0.018; BUT-B: *p* = 0.048), Supplementary Tables 9–10a do so for the BUT-B-PST and BUT-B-PSDI.

#### 3.2.7 Cut-off values

##### 3.2.7.1 Screening

Supplementary Table 3 shows that overall, the originally proposed cutoff score of >1.2 for the BUT-A corresponds to *T* > 53.5, which is close to the general population mean of *T* = 50 and represents an only mildly elevated score. The earlier cut-off values proposed (Cuzzolaro et al., 2006) were based on ROC analysis and provide an optimal balance of sensitivity and specificity. The ROC analysis of the present data indicated a cut-off value of RS > 1.62 as providing optimal sensitivity (92%) and specificity (64.6%).

##### 3.2.7.2 Reliable and clinically significant change

Supplementary Appendix 2 and Supplementary Table 12 present cut-off values for Clinical Significant Change and Reliable Change (Jacobson et al., 1999). These analyses suggest an RCI-95 of 0.41, which corresponds roughly to a shift in *T*-score of 5 points. Furthermore, the cut-off value for recovery for the GCC is RS > 1.77 (*T* > 57.1) for the BUT-A-GSI. Suggested cut-off values for recovery for the BUT-B-PST and BUT-B-PSDI were *RS* > 15.9 (*T* > 55.2) and *RS* >1.09 (*T* > 56.1), respectively.

## 4 Discussion

The aim of the present study was to psychometrically evaluate the BUT in the GCC. Reliability, validity, clinical utility were supported and the original five-factor structure of the BUT-A and the eight-factor structure of the BUT-B were confirmed in the combined clinical and community-based samples. Modification indices suggested that some items measure similar concepts, which should be considered when interpreting the factor structure: body ownership (feeling detached from your body, and feeling your body does not belong to you), self-observation (being in front of the mirror, and looking at oneself), and body parts: mustache-beard, stomach-abdomen, thighs-legs, buttocks-hips, and chest-breasts. These overlaps are likely due to linguistic/cultural adaptation, as they were not reported by Pokrajac-Bulian et al. (2015), who examined modification indices. Allowing correlated error terms between items improved model fit, and future work should review conceptually overlapping pairs and consider removal of redundant items. Consequently, future work could establish a shortened version, or conduct an Item Response Theory (IRT) analysis (De Beurs et al., 2022) to refine the scales. In addition, gender specific norms were established. Furthermore, sensitivity to change was confirmed in a small clinical subsample. Moreover, there was an overrepresentation of zero-responses, especially in the community-based sample. Subsequently, a limitation of the BUT may be that cultural minimization of body concerns and reluctance to endorse sensitive items can affect responses (Thompson et al., 2004). Therefore, it is necessary to utilize normalized *T*-scores for norm tables, because raw scores should first be normalized with a curvilinear conversion formula. Finally, zero-inflation could impact the factor analytic results. Future research could employ zero-inflated factor analytic techniques, such as Factor Mixture Modeling (Lubke and Muthen, 2005), Zero-Inflated IRT (De Boeck et al., 2011), or All-Zero Inflated Exploratory Factor Analysis (Flora and Curran, 2004), to explicitly account for the impact of structural zero-responses on factor loadings and model parameters.

The present study has several limitations. Although the BUT was available in Arabic and found understandable, all participants in the clinical sample preferred the English version over the Arabic one. This was in accordance with other work in the UAE (Melisse et al., 2025), and might be because the therapy sessions were mainly held in English, more modernized individuals were seeking therapy, most citizens being bilingual, social desirability or sample bias toward English speaking populations (Alkhadari et al., 2016; Griffiths et al., 2015; Gulf Articles, 2025). This preference could also reflect the dominance of English in health-care services in the UAE (Al-Yateem et al., 2023). Future studies should examine whether the language of administration of the BUT affects responses, if the BUT is equally suitable for less bilingual individuals and future validation in a purely Arab speaking sample is recommended. An important limitation was the absence of test–retest reliability, which prevented drawing conclusion of the stability of scores on the measure over a short test–retest interval. Consequently, assessing test–retest reliability is recommended for future research. Sensitivity to change was based on pre-posttreatment data of 20 participants only, since a small sample had completed treatment within the studies time window. Its interpretation warrants caution. Finally, generalizability might be limited due to underrepresentation of some GCC countries, self-selection from convenience sampling via social media, and a smaller male sample. Especially, norms for males should be interpreted with caution. A strength of the study was the large sample size, the more remarkable given the socially reclusive population (Al-Darmaki, 2003), and the implementation of app based use. Finally, this was the second study in the region including a clinical sample (el Khazen-Hadati et al., 2024), future work could benefit from cross-cultural comparisons, and integration into clinical practice.

In conclusion, the BUT shows strong promise as a valid assessment tool for use in the GCC, the original five-factor structure of the BUT-A and the eight-factor structure of the BUT-B were confirmed. However, item redundancy or item overlap should be reviewed, and raw scores should be normalized when utilizing the BUT norms.

## Data Availability

The raw data supporting the conclusions of this article will be made available by the authors, upon reasonable request.

## Generative AI sttaement

The authors declare that no Gen AI was used in the creation of this manuscript.

Any alternative text (alt text) provided alongside figures in this article has been generated by Frontiers with the support of artificial intelligence and reasonable efforts have been made to ensure accuracy, including review by the authors wherever possible. If you identify any issues, please contact us.
