# Supplementary material for: Psychometric evaluation of the body uneasiness test in the Arab Gulf region
Source: Front Psychol. 2025 Nov 14;16:1690293. doi: 10.3389/fpsyg.2025.1690293 (PMC12662080; doi:10.3389/fpsyg.2025.1690293)
Supplement: Supplementary file 1 [file Supplementary_file_1.docx]

**Supplementary Table 1**

*Basic psychometrics for BUT-A-items (N = 712)*

| Item | *M* | *SD* | *median* | *skewness* | *kurtosis* | 0 | 1 | 2 | 3 | 4 | 5 |
| --- | --- | --- | --- | --- | --- | --- | --- | --- | --- | --- | --- |
| 1 | 1.78 | 1.45 | 2 | 0.54 | -0.53 | 24 | 21 | 28 | 11 | 9 | 6 |
| 2 | 1.71 | 1.70 | 1 | 0.64 | -0.81 | 36 | 15 | 21 | 10 | 7 | 11 |
| 3 | 2.15 | 1.76 | 2 | 0.31 | -1.18 | 25 | 15 | 23 | 11 | 11 | 16 |
| 4 | 2.02 | 1.73 | 2 | 0.40 | -1.12 | 27 | 18 | 19 | 13 | 10 | 13 |
| 5 | 1.26 | 1.52 | 1 | **1.03** | -0.07 | 46 | 19 | 14 | 10 | 6 | 5 |
| 6 | 2.11 | 1.84 | 2 | 0.34 | **-1.27** | 29 | 14 | 19 | 11 | 9 | 18 |
| 7 | 1.24 | 1.54 | 1 | **1.07** | 0.07 | 49 | 16 | 16 | 9 | 5 | 6 |
| 8 | 1.06 | 1.46 | 0 | **1.28** | 0.55 | **55** | 15 | 13 | 8 | 5 | 4 |
| 9 | 2.30 | 1.95 | 2 | 0.20 | **-1.48** | 28 | 13 | 16 | 10 | 9 | 24 |
| 10 | 1.74 | 1.74 | 1 | 0.65 | -0.91 | 35 | 19 | 16 | 9 | 9 | 12 |
| 11 | 1.34 | 1.60 | 1 | 0.98 | -0.23 | 46 | 16 | 16 | 8 | 7 | 7 |
| 12 | 1.88 | 1.84 | 1 | 0.49 | **-1.20** | 36 | 15 | 14 | 10 | 10 | 15 |
| 13 | 1.01 | 1.43 | 0 | **1.28** | 0.59 | **57** | 13 | 13 | 8 | 4 | 4 |
| 14 | 1.18 | 1.60 | 0 | **1.17** | 0.16 | **55** | 12 | 13 | 9 | 4 | 8 |
| 15 | 0.94 | 1.43 | 0 | **1.48** | **1.18** | **60** | 13 | 12 | 6 | 4 | 5 |
| 16 | 0.81 | 1.38 | 0 | **1.62** | **1.50** | **67** | 10 | 9 | 7 | 4 | 3 |
| 17 | 1.10 | 1.46 | 0 | **1.25** | 0.54 | **52** | 16 | 16 | 6 | 5 | 5 |
| 18 | 1.69 | 1.88 | 1 | 0.65 | **-1.09** | 45 | 10 | 15 | 7 | 9 | 14 |
| 19 | 0.86 | 1.32 | 0 | **1.54** | 1.51 | **61** | 14 | 12 | 6 | 4 | 3 |
| 20 | 1.61 | 1.73 | 1 | 0.70 | -0.85 | 41 | 14 | 16 | 10 | 8 | 10 |
| 21 | 1.64 | 1.86 | 1 | 0.71 | -1 | 46 | 11 | 13 | 8 | 8 | 14 |
| 22 | 1.85 | 1.78 | 2 | 0.52 | -1.08 | 34 | 15 | 18 | 10 | 10 | 13 |
| 23 | 1.79 | 1.83 | 1 | 0.59 | -1.09 | 37 | 17 | 13 | 10 | 8 | 14 |
| 24 | 0.98 | 1.50 | 0 | **1.43** | 0.89 | **62** | 11 | 12 | 6 | 5 | 5 |
| 25 | 1.04 | 1.53 | 0 | **1.33** | 0.57 | **58** | 13 | 11 | 8 | 4 | 6 |
| 26 | 1.24 | 1.62 | 0 | **1.09** | -0.08 | **52** | 13 | 13 | 8 | 6 | 7 |
| 27 | 1.80 | 1.82 | 1 | 0.60 | **-1.03** | 37 | 15 | 17 | 10 | 6 | 15 |
| 28 | 1.07 | 1.54 | 0 | **1.28** | 0.44 | **58** | 12 | 12 | 7 | 5 | 6 |
| 29 | 1.87 | 1.44 | 0 | **1.61** | **1.45** | **65** | 10 | 10 | 6 | 4 | 5 |
| 30 | 1.00 | 1.52 | 0 | **1.36** | 0.63 | **62** | 10 | 11 | 7 | 5 | 5 |
| 31 | 1.58 | 1.73 | 1 | 0.70 | -0.88 | 43 | 14 | 14 | 11 | 10 | 9 |
| 32 | 2.09 | 1.87 | 2 | 0.31 | -1.34 | 32 | 12 | 16 | 13 | 10 | 17 |
| 33 | 1.74 | 1.78 | 1 | 0.59 | -1.01 | 39 | 12 | 18 | 10 | 8 | 12 |
| 34 | 1.46 | 1.79 | 1 | 0.90 | -0.66 | 49 | 13 | 13 | 6 | 8 | 12 |

*M* = Mean; *SD* = Standard deviation; Response option = Percentage of responses on response option.

Bold items have low endorsement.

Chronbach’s alpha = 0.98, Omega Hierarchical = .89.

**Supplementary Table 2**

*Basic psychometrics for BUT-B-items (N = 698)*

| Item | *M* | *SD* | *median* | *skewness* | *kurtosis* | 0 | 1 | 2 | 3 | 4 | 5 |
| --- | --- | --- | --- | --- | --- | --- | --- | --- | --- | --- | --- |
| 1 | 0.88 | 1.32 | 0 | **1.51** | **1.43** | **60** | 14 | 13 | 6 | 4 | 3 |
| 2 | 0.61 | 1.15 | 0 | **2.04** | **3.65** | **71** | 10 | 9 | 5 | 1 | 2 |
| 3 | 1.08 | 1.49 | 0 | **1.27** | 0.52 | **55** | 14 | 14 | 7 | 5 | 5 |
| 4 | 1.22 | 1.53 | 1 | **1.13** | 0.23 | 49 | 15 | 17 | 8 | 4 | 7 |
| 5 | 1.25 | 1.51 | 1 | 1.00 | -0.07 | 48 | 14 | 19 | 8 | 7 | 5 |
| 6 | 0.77 | 1.31 | 0 | **1.73** | **2.10** | **66** | 11 | 11 | 5 | 4 | 3 |
| 7 | 0.71 | 1.22 | 0 | **1.90** | **3.03** | **65** | 15 | 10 | 4 | 3 | 3 |
| 8 | 0.64 | 1.14 | 0 | **1.96** | **3.41** | **69** | 12 | 11 | 4 | 2 | 2 |
| 9 | 1.17 | 1.55 | 0 | **1.17** | 0.20 | **52** | 14 | 14 | 7 | 6 | 6 |
| 10 | 0.80 | 1.30 | 0 | **1.71** | **2.13** | **64** | 14 | 10 | 6 | 3 | 3 |
| 11 | 0.65 | 1.20 | 0 | **2.01** | **3.36** | **70** | 13 | 8 | 5 | 3 | 2 |
| 12 | 1.15 | 1.50 | 0 | **1.19** | 0.36 | **52** | 15 | 15 | 8 | 4 | 6 |
| 13 | 1.41 | 0.97 | 0 | **2.76** | **7.73** | **80** | 8 | 6 | 3 | 1 | 1 |
| 14 | 0.67 | 1.29 | 0 | **2.00** | **3.11** | **73** | 8 | 9 | 5 | 2 | 3 |
| 15 | 0.80 | 1.39 | 0 | **1.75** | **2.02** | **67** | 10 | 10 | 4 | 4 | 4 |
| 16 | 0.62 | 1.36 | 0 | **2.24** | **3.88** | **77** | 7 | 6 | 2 | 3 | 5 |
| 17 | 0.38 | 1.06 | 0 | **3.12** | **9.25** | **85** | 5 | 4 | 3 | 1 | 3 |
| 18 | 1.06 | 1.55 | 0 | **1.34** | 0.61 | **59** | 11 | 13 | 7 | 4 | 7 |
| 19 | 0.74 | 1.35 | 0 | **1.87** | **2.51** | **69** | 9 | 10 | 3 | 4 | 4 |
| 20 | 1.20 | 1.68 | 0 | **1.15** | -0.04 | **57** | 8 | 14 | 6 | 5 | 9 |
| 21 | 0.84 | 1.42 | 0 | **1.69** | **1.82** | **66** | 10 | 10 | 5 | 3 | 5 |
| 22 | 0.92 | 1.53 | 0 | **1.57** | **1.24** | **65** | 9 | 10 | 5 | 4 | 7 |
| 23 | 1.24 | 1.66 | 0 | **1.09** | -0.12 | **54** | 11 | 14 | 7 | 6 | 8 |
| 24 | 1.76 | 1.96 | 1 | 0.60 | **-1.22** | **46** | 9 | 12 | 9 | 7 | 18 |
| 25 | 1.75 | 1.92 | 1 | 0.59 | **-1.19** | **45** | 8 | 14 | 10 | 7 | 16 |
| 26 | 0.85 | 1.44 | 0 | **1.64** | **1.56** | **67** | 9 | 10 | 6 | 4 | 5 |
| 27 | 1.21 | 1.66 | 0 | **1.12** | -0.11 | **56** | 10 | 12 | 8 | 6 | 8 |
| 28 | 1.30 | 1.70 | 0 | 0.99 | -0.44 | **54** | 10 | 11 | 9 | 7 | 8 |
| 29 | 1.55 | 1.82 | 1 | 0.77 | -0.89 | 48 | 11 | 12 | 9 | 7 | 12 |
| 30 | 0.95 | 1.44 | 0 | **1.44** | **1.03** | **61** | 11 | 13 | 7 | 4 | 5 |
| 31 | 1.21 | 1.68 | 0 | **1.15** | -0.06 | **56** | 10 | 13 | 6 | 6 | 9 |
| 32 | 0.61 | 1.29 | 0 | **2.23** | **4.04** | **75** | 9 | 6 | 4 | 2 | 4 |
| 33 | 0.77 | 1.36 | 0 | **1.81** | **2.23** | **68** | 12 | 8 | 5 | 4 | 4 |
| 34 | 0.75 | 1.30 | 0 | **1.85** | **2.66** | **67** | 12 | 11 | 5 | 2 | 4 |
| 35 | 0.83 | 1.43 | 0 | **1.73** | **1.90** | **66** | 11 | 10 | 4 | 4 | 5 |
| 36 | 1.27 | 1.63 | 0 | **1.07** | -0.11 | **51** | 13 | 14 | 8 | 5 | 8 |
| 37 | 0.55 | 1.17 | 0 | **2.35** | **4.86** | **75** | 10 | 6 | 4 | 2 | 3 |

*M* = Mean; *SD* = Standard deviation; Response option = Percentage of responses on response option.

**Supplementary Table 2a**

*Means and SD for male and female respondents.*

| Scale | Males (*N*=86) | | Females (*N*=622) | |  |  |  |  |
| --- | --- | --- | --- | --- | --- | --- | --- | --- |
| BUT-A | *M* | *SD* | *M* | *SD* | *t* | *p* | *d* | *95%CI* |
| General Severity Index | 1.10 | 1.18 | 1.52 | 1.26 | 3.10 | .0012 | 0.34 | [0.11, 0.57] |
| Weight phobia | 1.20 | 1.28 | 1.79 | 1.45 | 3.96 | .0001 | 0.41 | [0.19, 0.64] |
| Body image concern | 1.33 | 1.38 | 1.64 | 1.43 | 1.91 | .0280 | 0.22 | [0.01, 0.45] |
| Avoidance | 0.81 | 1.12 | 1.15 | 1.20 | 2.55 | .0060 | 0.28 | [0.05, 0.51] |
| Compulsive self-monitoring | 1.06 | 1.13 | 1.59 | 1.30 | 3.98 | .0001 | 0.41 | [0.19, 0.64] |
| Depersonalization | 0.86 | 1.16 | 1.27 | 1.29 | 2.97 | .0018 | 0.32 | [0.09, 0.54] |
| BUT_B | *M* | *SD* | *M* | *SD* | *t* | *p* | *d* | *95%CI* |
| Positive Symptoms Total | 10.91 | 11.96 | 14.31 | 11.74 | 2.50 | 0.0063 | 0.29 | [0.06, 0.52] |
| Positive Symptom Distress Index | 0.65 | 0.81 | 0.99 | 0.98 | 3.52 | 0.0003 | 0.36 | [0.13, 0.58] |
| B1 Mouth | 0.46 | 0.73 | 0.91 | 1.05 | 4.93 | 0.0000 | 0.44 | [0.21, 0.66] |
| B2 Face Shape | 0.53 | 0.79 | 0.75 | 0.97 | 1.95 | 0.0257 | 0.23 | [0.00, 0.45] |
| B3 Thighs | 1.04 | 1.27 | 1.52 | 1.49 | 3.17 | 0.0010 | 0.32 | [0.10, 0.55] |
| B4 Legs | 0.54 | 0.91 | 0.90 | 1.09 | 3.32 | 0.0006 | 0.34 | [0.11, 0.57] |
| B5 Arms | 0.70 | 1.09 | 1.03 | 1.27 | 2.59 | 0.0054 | 0.27 | [0.04, 0.49] |
| B6 Moustache | 0.56 | 1.04 | 0.70 | 1.08 | 1.13 | 0.1299 | 0.13 | [0.10, 0.36] |
| B7 Skin | 0.85 | 1.22 | 1.29 | 1.34 | 3.10 | 0.0012 | 0.33 | [0.11, 0.56] |
| B8 Blushing | 0.66 | 0.90 | 0.96 | 1.06 | 2.79 | 0.0031 | 0.29 | [0.06, 0.51] |

A significant difference with higher scores for females was found on all scales except one (Moustache/hairs).

**Supplementary Table 2b**

*Means and SD for three age groups.*

| Scale | 16-20 (*N*=274) | | 21-23 (*N*=218) | | 24+ (*N*=220) | |  |  |  |  |
| --- | --- | --- | --- | --- | --- | --- | --- | --- | --- | --- |
| BUT-A | *M* | *SD* | *M* | *SD* | *M* | *SD* | *F* | *p* | *eta^2^* | *pc* |
| General Severity Index | 1.58 | 1.34 | 1.40 | 1.22 | 1.4 | 1.18 | 1.63 | 0.1978 | 0.005 |  |
| Weigth phobia | 1.82 | 1.51 | 1.57 | 1.37 | 1.75 | 1.41 | 1.93 | 0.1459 | 0.005 |  |
| Body image concern | 1.71 | 1.55 | 1.53 | 1.39 | 1.54 | 1.30 | 1.17 | 0.3112 | 0.004 |  |
| Avoidance | 1.18 | 1.26 | 1.04 | 1.18 | 1.07 | 1.12 | 0.94 | 0.3930 | 0.003 |  |
| Compulsive self-monitoring | 1.69 | 1.34 | 1.49 | 1.24 | 1.36 | 1.25 | 4.07 | 0.0174 | 0.011 | 1>3 |
| Depersonalization | 1.33 | 1.36 | 1.19 | 1.27 | 1.11 | 1.21 | 1.77 | 0.1703 | 0.005 |  |
|  | 16-20 (*N*=269) | | 21-23 (*N*=218) | | 24+ (*N*=211) | |  |  |  |  |
| BUT-B | *M* | *SD* | *M* | *SD* | *M* | *SD* | *F* | *p* | *eta^2^* | *pc* |
| Positive Symptoms Total | 15.27 | 12.08 | 13.51 | 12.22 | 12.54 | 10.89 | 3.46 | 0.0321 | 0.010 | 1>3 |
| Positive Symptom Distress Index | 1.10 | 1.04 | 0.90 | 0.95 | 0.81 | 0.84 | 5.64 | 0.0038 | 0.016 | 1>3 |
| B1 Mouth | 0.99 | 1.09 | 0.90 | 1.04 | 0.62 | 0.87 | 9.63 | 0.0001 | 0.024 | 1>3, 2>3 |
| B2 Face Shape | 0.88 | 1.05 | 0.69 | 0.89 | 0.55 | 0.85 | 7.05 | 0.0010 | 0.020 | 1>3 |
| B3 Thighs | 1.61 | 1.59 | 1.26 | 1.38 | 1.48 | 1.39 | 3.53 | 0.0302 | 0.010 | 1>2 |
| B4 Legs | 1.03 | 1.19 | 0.76 | 1.03 | 0.75 | 0.93 | 5.18 | 0.0060 | 0.016 | 1>2, 1>3 |
| B5 Arms | 1.15 | 1.37 | 0.95 | 1.24 | 0.83 | 1.09 | 4.04 | 0.0183 | 0.011 | 1>3 |
| B6 Moustache | 0.80 | 1.16 | 0.74 | 1.13 | 0.49 | 0.85 | 6.77 | 0.0013 | 0.015 | 1>3, 2>3 |
| B7 Skin | 1.33 | 1.42 | 1.25 | 1.32 | 1.10 | 1.22 | 1.92 | 0.1473 | 0.005 |  |
| B8 Blushing | 1.06 | 1.14 | 0.87 | 1.02 | 0.80 | 0.94 | 3.99 | 0.0191 | 0.012 | 1>3 |

Anova revealed a difference between the groups *F*(2,454.43) = 3.46; *p* = 0.032; Eta2 = 0.010; according to Tukey's HSD 1>3, group 16-20 and 24-72 differed (Cohen's *d* = 0.24, 95%CI [0.05 - 0.42]). General conclusion. For the BUT-A: there was only a significant difference between age groups on compulsive self-monitoring with more monitoring in the youngest group compared to the oldest group. However, BUT-B scores differed for most scales among age groups, with lower scores with rising age. Conclusion: no need to make separate crosswalk tables for age groups.

**Supplementary Table 3**

*Cross-Walk Table from Raw Scores to T- and PR-scores for General Severity Index*

| RS | T^1^ | PR_n^2^ | PR_cl^3^ |
| --- | --- | --- | --- |
| 0.00 | 35.0 | 6 | 0 |
| 0.03 | 37.6 | 10 | 0 |
| 0.06 | 38.7 | 13 | - |
| 0.09 | 39.6 | 15 | - |
| 0.12 | 40.3 | 18 | - |
| 0.15 | 41.0 | 20 | 2 |
| 0.18 | 41.6 | 22 | 2 |
| 0.21 | 42.1 | 23 | - |
| 0.24 | 42.7 | 25 | - |
| 0.26 | 43.0 | 26 | - |
| 0.29 | 43.5 | 28 | - |
| 0.32 | 43.9 | 30 | 4 |
| 0.35 | 44.4 | 31 | 5 |
| 0.38 | 44.8 | 32 | - |
| 0.41 | 45.2 | 34 | - |
| 0.44 | 45.6 | 35 | 6 |
| 0.47 | 45.9 | 37 | 7 |
| 0.50 | 46.3 | 38 | 7 |
| 0.53 | 46.7 | 39 | - |
| 0.56 | 47.0 | 40 | - |
| 0.59 | 47.4 | 42 | 9 |
| 0.62 | 47.7 | 43 | - |
| 0.65 | 48.0 | 44 | 10 |
| 0.68 | 48.3 | 45 | 10 |
| 0.71 | 48.6 | 46 | 11 |
| 0.74 | 48.9 | 47 | 11 |
| 0.76 | 49.1 | 48 | 12 |
| 0.79 | 49.4 | 49 | - |
| 0.82 | 49.7 | 50 | - |
| 0.85 | 50.0 | 51 | 13 |
| 0.88 | 50.3 | 52 | - |
| 0.91 | 50.6 | 53 | 14 |
| 0.94 | 50.8 | 54 | 15 |
| 0.97 | 51.1 | 54 | 16 |
| 1.00 | 51.4 | 55 | 16 |
| 1.03 | 51.6 | 56 | - |
| 1.06 | 51.9 | 57 | - |
| 1.09 | 52.1 | 58 | 18 |
| 1.12 | 52.4 | 59 | 18 |
| 1.15 | 52.6 | 59 | 19 |
| 1.18 | 52.9 | 60 | - |
| 1.21 | 53.1 | 61 | - |
| 1.24 | 53.4 | 62 | - |
| 1.26 | 53.5 | 62 | 21 |
| 1.29 | 53.7 | 63 | 22 |
| 1.32 | 54.0 | 64 | 22 |
| 1.35 | 54.2 | 65 | - |
| 1.38 | 54.4 | 65 | 23 |
| 1.41 | 54.7 | 66 | - |
| 1.44 | 54.9 | 67 | 25 |
| 1.47 | 55.1 | 67 | - |
| 1.50 | 55.3 | 68 | 26 |
| 1.53 | 55.5 | 69 | - |
| 1.56 | 55.7 | 69 | 27 |
| 1.59 | 56.0 | 70 | - |
| 1.62 | 56.2 | 70 | 28 |
| 1.65 | 56.4 | 71 | 29 |
| 1.68 | 56.6 | 72 | 29 |
| 1.71 | 56.8 | 72 | 30 |
| 1.74 | 57.0 | 73 | 31 |
| 1.76 | 57.1 | 73 | 31 |
| 1.79 | 57.3 | 74 | 32 |
| 1.82 | 57.5 | 74 | - |
| 1.85 | 57.7 | 75 | 33 |
| 1.88 | 57.9 | 75 | 34 |
| 1.91 | 58.1 | 76 | 34 |
| 1.94 | 58.3 | 77 | - |
| 1.97 | 58.5 | 77 | 36 |
| 2.00 | 58.7 | 78 | 36 |
| 2.03 | 58.9 | 78 | 37 |
| 2.06 | 59.1 | 79 | - |
| 2.09 | 59.2 | 79 | 38 |
| 2.12 | 59.4 | 80 | 39 |
| 2.15 | 59.6 | 80 | 40 |
| 2.18 | 59.8 | 81 | 40 |
| 2.21 | 60.0 | 81 | - |
| 2.24 | 60.2 | 81 | - |
| 2.26 | 60.3 | 82 | 42 |
| 2.29 | 60.5 | 82 | 43 |
| 2.32 | 60.6 | 83 | 44 |
| 2.35 | 60.8 | 83 | 44 |
| 2.38 | 61.0 | 84 | - |
| 2.41 | 61.2 | 84 | - |
| 2.44 | 61.3 | 84 | - |
| 2.47 | 61.5 | 85 | 47 |
| 2.50 | 61.7 | 85 | 48 |
| 2.53 | 61.8 | 86 | - |
| 2.56 | 62.0 | 86 | 49 |
| 2.59 | 62.2 | 86 | - |
| 2.62 | 62.3 | 87 | - |
| 2.65 | 62.5 | 87 | - |
| 2.68 | 62.7 | 88 | 52 |
| 2.71 | 62.8 | 88 | 53 |
| 2.74 | 63.0 | 88 | - |
| 2.76 | 63.1 | 89 | 54 |
| 2.79 | 63.3 | 89 | 55 |
| 2.82 | 63.4 | 89 | 56 |
| 2.85 | 63.6 | 90 | 57 |
| 2.88 | 63.8 | 90 | 57 |
| 2.91 | 63.9 | 90 | 58 |
| 2.94 | 64.1 | - | 59 |
| 2.97 | 64.2 | - | 60 |
| 3.00 | 64.4 | 91 | - |
| 3.03 | 64.6 | - | 61 |
| 3.06 | 64.7 | - | 62 |
| 3.09 | 64.9 | - | 63 |
| 3.12 | 65.0 | 93 | - |
| 3.15 | 65.2 | 93 | 64 |
| 3.18 | 65.3 | 93 | 65 |
| 3.21 | 65.5 | 94 | - |
| 3.24 | 65.6 | - | 67 |
| 3.26 | 65.7 | - | 67 |
| 3.29 | 65.9 | - | 68 |
| 3.32 | 66.0 | 95 | 69 |
| 3.35 | 66.2 | - | 70 |
| 3.38 | 66.3 | 95 | 71 |
| 3.41 | 66.5 | - | 72 |
| 3.44 | 66.6 | - | 72 |
| 3.47 | 66.8 | - | 73 |
| 3.50 | 66.9 | 96 | 74 |
| 3.53 | 67.1 | - | 75 |
| 3.56 | 67.2 | - | 76 |
| 3.59 | 67.3 | - | 77 |
| 3.62 | 67.5 | - | 78 |
| 3.65 | 67.6 | - | 78 |
| 3.68 | 67.8 | - | - |
| 3.71 | 67.9 | - | 80 |
| 3.74 | 68.1 | 98 | 81 |
| 3.76 | 68.2 | 99 | 82 |
| 3.79 | 68.3 | - | 82 |
| 3.82 | 68.4 | - | - |
| 3.85 | 68.6 | 99 | 84 |
| 3.88 | 68.7 | 100 | 85 |
| 3.91 | 68.9 | - | 86 |
| 3.94 | 69.0 | 100 | 87 |
| 3.97 | 69.1 | - | 88 |
| 4.00 | 69.3 | - | 88 |
| 4.03 | 69.4 | - | - |
| 4.06 | 69.5 | - | 90 |
| 4.09 | 69.7 | - | 91 |
| 4.12 | 69.8 | 100 | 92 |
| 4.15 | 69.9 | - | 92 |
| 4.18 | 70.1 | - | 93 |
| 4.21 | 70.2 | - | 94 |
| 4.24 | 70.4 | - | - |
| 4.26 | 70.4 | 100 | 95 |
| 4.29 | 70.6 | - | 95 |
| 4.32 | 70.7 | - | - |
| 4.35 | 70.8 | 100 | 96 |
| 4.38 | 71.0 | - | - |
| 4.41 | 71.1 | - | 97 |
| 4.44 | 71.2 | - | 97 |
| 4.47 | 71.4 | - | - |
| 4.50 | 71.5 | - | - |
| 4.53 | 71.6 | 100 | - |
| 4.56 | 71.8 | - | - |
| 4.59 | 71.9 | - | - |
| 4.62 | 72.0 | - | 98 |
| 4.65 | 72.1 | 100 | - |
| 4.68 | 72.3 | - | - |
| 4.71 | 72.4 | 100 | - |
| 4.74 | 72.5 | - | 99 |
| 4.76 | 72.6 | - | - |
| 4.79 | 72.7 | - | - |
| 4.82 | 72.9 | - | - |
| 4.85 | 73.0 | - | 99 |
| 4.88 | 73.1 | - | - |
| 4.91 | 73.2 | - | - |
| 4.94 | 73.4 | - | - |
| 4.97 | 73.5 | - | - |
| 5.00 | 73.6 | - | - |
| NB: RS = Raw Score; T = T-score; PR_n = Percentile Rank score general population; PR_cl = Percentile Rank score clinical sample. | | | |
| ^1^Formula for population all (ID:17) for RS->TRankit: y=35.0+18830.432*(1-exp(-exp(5.344e-01*(ln(x+.0001)-ln(536614.983))))); a Weib2 function. | | | |
| ^2^Formula for population all (ID:17) for RS->PR_n: y=6.4+117.849*(1-exp(-exp(7.892e-01*(ln(x+.0001)-ln(2.202))))); a Weib2 function. | | | |
| ^3^Formula for clinical all (ID:13) for RS->PR_cl: y=-89.8+188.876/((1+exp(-6.561*(x-4.312)))^2.662e-02); a sigm-L5 function.  *Note.* The originally proposed cutoff score (>1.2) for the BUT-A (Cuzzolaro et al., 2006) corresponds to a *T*-score of >53.5, which is ~0.35 standard deviations above the population mean of *T* = 50, indicating a mildly elevated but not significant score. The original cutoff (>1.2) was selected via ROC analysis to optimize sensitivity and specificity, but the exact figures were not reported. In contrast, the present data identified a slightly higher cutoff (RS >1.62), resulting in a sensitivity of 92% and specificity of 64.6%. This comparison highlights that the newly derived cutoff favors sensitivity at the expense of some specificity, and the *T*-score interpretation clarifies the clinical significance.  During treatment, the cut-off value for recovery for the GCC is RS>1.77 (*T* >57.1) for the BUT-A-GSI. Suggested cut-off values for recovery for the BUT-B-PST and BUT-B-PSDI were *RS* > 15.9 (*T* >55.2) and *RS* >1.09 (*T* >56.1), respectively. The present clinical example illustrates the change in standardized scores from pre- to post-intervention: A clinical case illustrated a decrease from a pretest *T*-score of 65 (corresponding PR-score ≈ 3.1; only 2.5% of the general population would score higher, indicating a clinical score) to a posttest *T*-score of 54 (PR-score ≈ 1.3; now 45% of the population scores higher), which indicates recovery, representing an improvement of ~1 SD and a normalization of symptoms. | | | |

**Supplementary Table 3a**

*Cross-Walk Table from Raw Scores to T- and PR-scores for General Severity Index for females and males*

|  | female | | | male | | |
| --- | --- | --- | --- | --- | --- | --- |
| RS | T1^1^ | PR_n1^2^ | PR_cl1^3^ | T2^4^ | PR_n2^5^ | PR_cl2^6^ |
| 0.00 | 34.7 | 6 | - | 37.3 | 10 | 3 |
| 0.03 | 37.0 | 9 | 0 | 40.5 | - | - |
| 0.06 | 38.1 | 12 | - | 41.9 | - | - |
| 0.09 | 39.0 | 14 | - | 42.9 | 22 | - |
| 0.12 | 39.7 | 16 | - | 43.8 | 25 | - |
| 0.15 | 40.4 | 18 | 2 | 44.6 | 28 | - |
| 0.18 | 41.0 | 20 | 2 | 45.2 | 31 | - |
| 0.21 | 41.5 | 22 | - | 45.9 | 33 | - |
| 0.24 | 42.1 | 23 | - | 46.4 | 36 | - |
| 0.26 | 42.4 | 25 | - | 46.8 | 37 | - |
| 0.29 | 42.9 | 26 | - | 47.3 | 39 | - |
| 0.32 | 43.3 | 28 | 4 | 47.8 | 41 | - |
| 0.35 | 43.7 | 29 | 5 | 48.3 | 43 | - |
| 0.38 | 44.2 | 30 | - | 48.7 | - | - |
| 0.41 | 44.6 | 32 | - | 49.2 | - | - |
| 0.44 | 45.0 | 33 | 6 | 49.6 | - | - |
| 0.47 | 45.3 | 34 | 7 | 50.0 | 51 | - |
| 0.50 | 45.7 | 36 | 7 | 50.3 | 52 | - |
| 0.53 | 46.1 | 37 | - | 50.7 | - | - |
| 0.56 | 46.4 | 38 | - | 51.1 | 55 | - |
| 0.59 | 46.7 | 39 | 8 | 51.4 | - | - |
| 0.62 | 47.1 | 40 | - | 51.7 | - | - |
| 0.65 | 47.4 | 42 | 9 | 52.1 | 59 | - |
| 0.68 | 47.7 | 43 | 10 | 52.4 | 61 | - |
| 0.71 | 48.0 | 44 | - | 52.7 | 62 | 8 |
| 0.74 | 48.3 | 45 | 11 | 53.0 | - | - |
| 0.76 | 48.5 | 46 | 11 | 53.2 | 64 | - |
| 0.79 | 48.8 | 47 | - | 53.5 | 65 | - |
| 0.82 | 49.1 | 48 | - | 53.8 | - | - |
| 0.85 | 49.4 | 49 | 13 | 54.0 | 67 | - |
| 0.88 | 49.7 | 49 | - | 54.3 | - | - |
| 0.91 | 50.0 | 50 | 14 | 54.6 | 69 | 17 |
| 0.94 | 50.3 | 51 | 14 | 54.8 | - | - |
| 0.97 | 50.5 | 52 | 15 | 55.1 | 71 | - |
| 1.00 | 50.8 | 53 | 15 | 55.4 | 72 | - |
| 1.03 | 51.1 | 54 | - | 55.6 | - | - |
| 1.06 | 51.3 | 55 | - | 55.8 | - | - |
| 1.09 | 51.6 | 56 | - | 56.1 | 74 | 25 |
| 1.12 | 51.8 | 57 | 17 | 56.3 | - | - |
| 1.15 | 52.1 | 57 | 18 | 56.6 | 75 | 28 |
| 1.18 | 52.3 | 58 | - | 56.8 | - | - |
| 1.21 | 52.6 | 59 | - | 57.0 | 77 | - |
| 1.24 | 52.8 | 60 | - | 57.2 | - | - |
| 1.26 | 53.0 | 60 | 20 | 57.4 | 78 | - |
| 1.29 | 53.2 | 61 | 20 | 57.6 | - | - |
| 1.32 | 53.4 | 62 | 21 | 57.8 | - | - |
| 1.35 | 53.7 | 63 | - | 58.0 | - | - |
| 1.38 | 53.9 | 63 | 22 | 58.2 | - | - |
| 1.41 | 54.1 | 64 | - | 58.4 | - | - |
| 1.44 | 54.4 | 65 | 23 | 58.6 | - | - |
| 1.47 | 54.6 | 65 | - | 58.8 | - | - |
| 1.50 | 54.8 | 66 | 24 | 59.0 | - | - |
| 1.53 | 55.0 | 67 | - | 59.2 | - | - |
| 1.56 | 55.2 | 67 | 25 | 59.4 | 83 | 42 |
| 1.59 | 55.5 | 68 | - | 59.6 | - | - |
| 1.62 | 55.7 | 69 | 27 | 59.8 | - | - |
| 1.65 | 55.9 | 69 | 27 | 60.0 | - | - |
| 1.68 | 56.1 | 70 | 28 | 60.2 | - | - |
| 1.71 | 56.3 | 71 | - | 60.3 | - | 47 |
| 1.74 | 56.5 | 71 | 29 | 60.5 | - | - |
| 1.76 | 56.6 | 72 | 29 | 60.6 | - | - |
| 1.79 | 56.9 | 72 | 30 | 60.8 | 86 | - |
| 1.82 | 57.1 | 73 | - | 61.0 | - | - |
| 1.85 | 57.3 | 73 | 31 | 61.2 | - | - |
| 1.88 | 57.5 | 74 | 32 | 61.3 | - | - |
| 1.91 | 57.7 | 75 | 33 | 61.5 | - | - |
| 1.94 | 57.9 | 75 | - | 61.7 | - | - |
| 1.97 | 58.0 | 76 | 34 | 61.8 | - | - |
| 2.00 | 58.2 | 76 | - | 62.0 | - | 54 |
| 2.03 | 58.4 | 77 | 35 | 62.2 | - | - |
| 2.06 | 58.6 | 77 | - | 62.3 | 89 | - |
| 2.09 | 58.8 | 78 | 37 | 62.5 | 89 | - |
| 2.12 | 59.0 | 78 | 37 | 62.6 | - | - |
| 2.15 | 59.2 | 79 | 38 | 62.8 | - | - |
| 2.18 | 59.4 | 79 | - | 63.0 | - | 58 |
| 2.21 | 59.6 | 80 | - | 63.1 | - | - |
| 2.24 | 59.8 | 80 | - | 63.3 | - | - |
| 2.26 | 59.9 | 81 | 40 | 63.4 | - | - |
| 2.29 | 60.1 | 81 | 41 | 63.5 | - | - |
| 2.32 | 60.2 | 82 | 42 | 63.7 | - | - |
| 2.35 | 60.4 | 82 | 42 | 63.8 | - | - |
| 2.38 | 60.6 | 82 | - | 64.0 | - | - |
| 2.41 | 60.8 | 83 | - | 64.1 | - | - |
| 2.44 | 61.0 | 83 | - | 64.2 | - | - |
| 2.47 | 61.1 | 84 | 45 | 64.4 | 92 | - |
| 2.50 | 61.3 | - | 46 | 64.5 | 92 | - |
| 2.53 | 61.5 | 85 | - | 64.7 | - | - |
| 2.56 | 61.7 | 85 | 48 | 64.8 | - | - |
| 2.59 | 61.8 | 86 | - | 65.0 | 92 | - |
| 2.62 | 62.0 | 86 | - | 65.1 | 92 | - |
| 2.65 | 62.2 | 86 | - | 65.2 | 93 | - |
| 2.68 | 62.3 | 87 | 51 | 65.4 | - | - |
| 2.71 | 62.5 | 87 | 51 | 65.5 | - | - |
| 2.74 | 62.7 | 88 | - | 65.6 | - | - |
| 2.76 | 62.8 | 88 | 53 | 65.7 | - | - |
| 2.79 | 63.0 | 88 | 53 | 65.9 | - | - |
| 2.82 | 63.1 | 89 | 54 | 66.0 | - | - |
| 2.85 | 63.3 | 89 | - | 66.1 | - | 71 |
| 2.88 | 63.5 | 89 | 56 | 66.2 | - | - |
| 2.91 | 63.6 | 90 | 57 | 66.4 | - | - |
| 2.94 | 63.8 | - | 57 | 66.5 | - | - |
| 2.97 | 63.9 | - | 58 | 66.6 | - | 73 |
| 3.00 | 64.1 | 91 | - | 66.8 | 94 | - |
| 3.03 | 64.3 | - | 60 | 66.9 | - | - |
| 3.06 | 64.4 | - | 61 | 67.0 | - | - |
| 3.09 | 64.6 | - | 61 | 67.1 | - | - |
| 3.12 | 64.7 | 92 | - | 67.2 | - | - |
| 3.15 | 64.9 | 93 | 63 | 67.4 | - | - |
| 3.18 | 65.1 | 93 | 64 | 67.5 | - | 76 |
| 3.21 | 65.2 | 93 | - | 67.6 | - | - |
| 3.24 | 65.4 | - | 66 | 67.7 | - | - |
| 3.26 | 65.5 | - | 66 | 67.8 | - | - |
| 3.29 | 65.6 | - | 67 | 67.9 | - | - |
| 3.32 | 65.8 | 94 | 68 | 68.0 | - | - |
| 3.35 | 65.9 | - | 69 | 68.2 | - | - |
| 3.38 | 66.1 | 95 | 70 | 68.3 | - | - |
| 3.41 | 66.2 | - | 71 | 68.4 | - | - |
| 3.44 | 66.4 | - | 72 | 68.5 | - | 80 |
| 3.47 | 66.5 | - | 72 | 68.6 | - | - |
| 3.50 | 66.7 | 96 | 73 | 68.7 | - | - |
| 3.53 | 66.9 | - | 74 | 68.8 | - | - |
| 3.56 | 67.0 | - | 75 | 69.0 | - | - |
| 3.59 | 67.1 | - | 76 | 69.1 | - | - |
| 3.62 | 67.3 | - | 77 | 69.2 | - | - |
| 3.65 | 67.4 | - | 78 | 69.3 | - | - |
| 3.68 | 67.6 | - | - | 69.4 | - | - |
| 3.71 | 67.7 | - | 80 | 69.5 | - | - |
| 3.74 | 67.9 | 99 | 81 | 69.6 | - | - |
| 3.76 | 68.0 | 99 | 81 | 69.7 | - | - |
| 3.79 | 68.1 | - | 82 | 69.8 | - | - |
| 3.82 | 68.3 | - | - | 69.9 | - | - |
| 3.85 | 68.4 | 100 | 84 | 70.0 | 96 | - |
| 3.88 | 68.6 | 100 | 85 | 70.1 | - | - |
| 3.91 | 68.7 | - | 86 | 70.2 | - | - |
| 3.94 | 68.8 | 100 | - | 70.3 | - | 86 |
| 3.97 | 69.0 | - | 88 | 70.4 | - | - |
| 4.00 | 69.1 | - | 89 | 70.5 | - | - |
| 4.03 | 69.3 | - | - | 70.6 | - | - |
| 4.06 | 69.4 | - | 90 | 70.7 | - | - |
| 4.09 | 69.6 | - | 91 | 70.8 | - | - |
| 4.12 | 69.7 | 100 | 92 | 70.9 | - | - |
| 4.15 | 69.8 | - | - | 71.0 | - | 88 |
| 4.18 | 70.0 | - | 93 | 71.1 | - | - |
| 4.21 | 70.1 | - | 94 | 71.2 | - | - |
| 4.24 | 70.2 | - | - | 71.3 | - | - |
| 4.26 | 70.3 | 100 | 95 | 71.4 | - | - |
| 4.29 | 70.5 | - | 95 | 71.5 | - | - |
| 4.32 | 70.6 | - | - | 71.6 | - | - |
| 4.35 | 70.8 | 100 | 96 | 71.7 | - | - |
| 4.38 | 70.9 | - | - | 71.8 | - | - |
| 4.41 | 71.0 | - | 97 | 71.9 | - | - |
| 4.44 | 71.2 | - | 97 | 72.0 | - | - |
| 4.47 | 71.3 | - | - | 72.1 | - | - |
| 4.50 | 71.4 | - | - | 72.2 | - | - |
| 4.53 | 71.6 | 100 | - | 72.3 | - | - |
| 4.56 | 71.7 | - | - | 72.4 | - | - |
| 4.59 | 71.8 | - | - | 72.5 | - | - |
| 4.62 | 72.0 | - | - | 72.6 | - | 93 |
| 4.65 | 72.1 | 100 | - | 72.6 | - | - |
| 4.68 | 72.2 | - | - | 72.7 | - | - |
| 4.71 | 72.4 | 100 | - | 72.8 | - | - |
| 4.74 | 72.5 | - | 99 | 72.9 | - | - |
| 4.76 | 72.6 | - | - | 73.0 | - | - |
| 4.79 | 72.7 | - | - | 73.1 | - | - |
| 4.82 | 72.8 | - | - | 73.2 | - | - |
| 4.85 | 73.0 | - | 99 | 73.3 | - | - |
| 4.88 | 73.1 | - | - | 73.3 | - | - |
| 4.91 | 73.2 | - | - | 73.4 | - | - |
| 4.94 | 73.4 | - | - | 73.5 | - | - |
| 4.97 | 73.5 | - | - | 73.6 | - | - |
| 5.00 | 73.6 | - | - | 73.7 | - | - |
| NB: RS = Raw Score; T = T-score; PR_n = Percentile Rank score general population; PR_cl = Percentile Rank score clinical sample. | | | | | | |
| ^1^Formula for population female (ID:17) for RS->TRankit: y=34.7+22050.029*(1-exp(-exp(5.487e-01*(ln(x+.0001)-ln(520390.273))))); a Weib2 function. | | | | | | |
| ^2^Formula for population female (ID:17) for RS->PR_n: y=6.0+122.723*(1-exp(-exp(8.062e-01*(ln(x+.0001)-ln(2.456))))); a Weib2 function. | | | | | | |
| ^3^Formula for clinical female (ID:13) for RS->PR_cl: y=-60.4+159.295/((1+exp(-6.353*(x-4.263)))^3.600e-02); a sigm-L5 function. | | | | | | |
| ^4^Formula for population male (ID:17) for RS->TRankit: y=37.3+97.992*(1-exp(-exp(5.120e-01*(ln(x+.0001)-ln(22.345))))); a Weib2 function. | | | | | | |
| ^5^Formula for population male (ID:17) for RS->PR_n: y=10.3+87.316*(1-exp(-exp(8.859e-01*(ln(x+.0001)-ln(8.073e-01))))); a Weib2 function. | | | | | | |
| ^6^Formula for clinical male (ID:18) for RS->PR_cl: y=8150.7-8147.819/((1+(x/6.798e-01)^9.162)^6.367e-04); a logis5 function. | | | | | | |

**Supplementary Table 4**

*Cross-Walk Table from Raw Scores to T- and PR-scores for Weight Phobia*

| RS | T^1^ | PR_n^2^ | PR_cl^3^ |
| --- | --- | --- | --- |
| 0.00 | 37.6 | 10 | 1 |
| 0.12 | 40.6 | 19 | - |
| 0.25 | 42.5 | 25 | 3 |
| 0.38 | 44.0 | 30 | 4 |
| 0.50 | 45.3 | 35 | 6 |
| 0.62 | 46.4 | 39 | 7 |
| 0.75 | 47.6 | 43 | 8 |
| 0.88 | 48.7 | 47 | 10 |
| 1.00 | 49.6 | 50 | 11 |
| 1.12 | 50.5 | 53 | 13 |
| 1.25 | 51.5 | 56 | 14 |
| 1.38 | 52.4 | 59 | 16 |
| 1.50 | 53.2 | 62 | 18 |
| 1.62 | 54.1 | 64 | 19 |
| 1.75 | 54.9 | 67 | 21 |
| 1.88 | 55.7 | 69 | 24 |
| 2.00 | 56.5 | 71 | 26 |
| 2.12 | 57.2 | 73 | 28 |
| 2.25 | 58.0 | 76 | 30 |
| 2.38 | 58.7 | 78 | 32 |
| 2.50 | 59.4 | 79 | 35 |
| 2.62 | 60.1 | 81 | 37 |
| 2.75 | 60.8 | 83 | 40 |
| 2.88 | 61.5 | 85 | 43 |
| 3.00 | 62.2 | 86 | 46 |
| 3.12 | 62.8 | 88 | 49 |
| 3.25 | 63.5 | 89 | 52 |
| 3.38 | 64.2 | 91 | 56 |
| 3.50 | 64.8 | 92 | 59 |
| 3.62 | 65.4 | 93 | 62 |
| 3.75 | 66.0 | 95 | 66 |
| 3.88 | 66.7 | 96 | 70 |
| 4.00 | 67.3 | 97 | 74 |
| 4.12 | 67.8 | 98 | 78 |
| 4.25 | 68.5 | 99 | 82 |
| 4.38 | 69.1 | 100 | 85 |
| 4.50 | 69.6 | 100 | 89 |
| 4.62 | 70.2 | 100 | 92 |
| 4.75 | 70.8 | - | 95 |
| 4.88 | 71.4 | 100 | 97 |
| 5.00 | 71.9 | 100 | 99 |
| NB: RS = Raw Score; T = T-score; PR_n = Percentile Rank score general population; PR_cl = Percentile Rank score clinical sample. | | | |
| ^1^Formula for population all (ID:17) for RS->TRankit: y=37.6+44880.990*(1-exp(-exp(6.534e-01*(ln(x+.0001)-ln(294742.638))))); a Weib2 function. | | | |
| ^2^Formula for population all (ID:17) for RS->PR_n: y=10.4+126.103*(1-exp(-exp(8.158e-01*(ln(x+.0001)-ln(3.325))))); a Weib2 function. | | | |
| ^3^Formula for clinical all (ID:13) for RS->PR_cl: y=-24.2+127.284/((1+exp(-3.472*(x-4.743)))^9.865e-02); a sigm-L5 function. | | | |

**Supplementary Table 4a**

*Cross-Walk Table from Raw Scores to T- and PR-scores for Weight Phobia for females and males*

|  | female | | | male | | |
| --- | --- | --- | --- | --- | --- | --- |
| RS | T1^1^ | PR_n1^2^ | PR_cl1^3^ | T2^4^ | PR_n2^5^ | PR_cl2^6^ |
| 0.00 | 37.3 | 10 | 1 | 39.6 | 15 | 0 |
| 0.12 | 40.1 | 17 | - | 44.5 | 28 | - |
| 0.25 | 41.8 | 23 | 3 | 46.8 | 38 | - |
| 0.38 | 43.3 | 28 | 4 | 48.5 | 45 | - |
| 0.50 | 44.6 | 32 | 5 | 49.9 | 50 | - |
| 0.62 | 45.7 | 36 | 6 | 51.1 | 55 | - |
| 0.75 | 46.9 | 40 | 8 | 52.3 | 60 | 12 |
| 0.88 | 47.9 | 44 | 9 | 53.5 | 64 | 15 |
| 1.00 | 48.9 | 47 | 10 | 54.4 | 67 | - |
| 1.12 | 49.8 | 50 | 12 | 55.3 | 70 | 20 |
| 1.25 | 50.8 | 54 | 13 | 56.3 | 73 | - |
| 1.38 | 51.7 | 57 | 15 | 57.2 | 76 | - |
| 1.50 | 52.6 | 59 | 16 | 57.9 | 78 | 29 |
| 1.62 | 53.4 | 62 | 18 | 58.7 | 80 | 32 |
| 1.75 | 54.3 | 65 | 20 | 59.5 | - | 35 |
| 1.88 | 55.1 | 67 | 22 | 60.2 | 84 | 38 |
| 2.00 | 55.9 | 70 | 24 | 60.9 | 85 | 41 |
| 2.12 | 56.6 | 72 | 26 | 61.6 | - | - |
| 2.25 | 57.4 | 74 | 28 | 62.3 | - | - |
| 2.38 | 58.2 | 76 | 30 | 63.0 | - | - |
| 2.50 | 58.9 | 78 | 33 | 63.6 | 90 | - |
| 2.62 | 59.6 | 80 | 35 | 64.2 | 91 | 55 |
| 2.75 | 60.3 | 82 | 38 | 64.8 | 92 | - |
| 2.88 | 61.1 | 84 | 41 | 65.4 | 93 | - |
| 3.00 | 61.7 | 85 | 44 | 66.0 | - | - |
| 3.12 | 62.4 | 87 | 47 | 66.5 | 95 | 67 |
| 3.25 | 63.1 | 88 | 50 | 67.1 | - | 70 |
| 3.38 | 63.8 | 90 | 54 | 67.7 | - | - |
| 3.50 | 64.4 | 91 | 57 | 68.2 | 97 | - |
| 3.62 | 65.1 | 93 | 61 | 68.7 | - | - |
| 3.75 | 65.7 | 94 | 65 | 69.2 | 98 | 80 |
| 3.88 | 66.4 | 96 | 69 | 69.8 | - | - |
| 4.00 | 67.0 | 97 | 73 | 70.2 | - | - |
| 4.12 | 67.6 | 98 | 76 | 70.7 | - | - |
| 4.25 | 68.3 | 99 | 81 | 71.2 | - | 90 |
| 4.38 | 68.9 | - | 85 | 71.7 | 100 | 92 |
| 4.50 | 69.5 | 100 | 88 | 72.2 | - | - |
| 4.62 | 70.1 | 100 | 91 | 72.6 | - | - |
| 4.75 | 70.7 | - | 94 | 73.1 | - | - |
| 4.88 | 71.3 | 100 | 97 | 73.6 | - | - |
| 5.00 | 71.9 | 100 | 98 | 74.0 | - | - |
| NB: RS = Raw Score; T = T-score; PR_n = Percentile Rank score general population; PR_cl = Percentile Rank score clinical sample. | | | | | | |
| ^1^Formula for population female (ID:17) for RS->TRankit: y=37.3+53745.697*(1-exp(-exp(6.789e-01*(ln(x+.0001)-ln(251459.664))))); a Weib2 function. | | | | | | |
| ^2^Formula for population female (ID:17) for RS->PR_n: y=9.9+127.448*(1-exp(-exp(8.593e-01*(ln(x+.0001)-ln(3.420))))); a Weib2 function. | | | | | | |
| ^3^Formula for clinical female (ID:13) for RS->PR_cl: y=-15.6+120.199/((1+exp(-2.843*(x-4.700)))^1.459e-01); a sigm-L5 function. | | | | | | |
| ^4^Formula for population male (ID:17) for RS->TRankit: y=39.6+6874.504*(1-exp(-exp(5.244e-01*(ln(x+.0001)-ln(120993.948))))); a Weib2 function. | | | | | | |
| ^5^Formula for population male (ID:17) for RS->PR_n: y=14.9+90.158*(1-exp(-exp(7.945e-01*(ln(x+.0001)-ln(1.192))))); a Weib2 function. | | | | | | |
| ^6^Formula for clinical male (ID:16) for RS->PR_cl: y=155.571*(1-exp(-exp(1.379*(ln(x+.0001)-ln(4.742))))); a Weib1 function. | | | | | | |

**Supplementary Table 5**

*Cross-Walk Table from Raw Scores to T- and PR-scores for Body Image Concern*

| RS | T^1^ | PR_n^2^ | PR_cl^3^ |
| --- | --- | --- | --- |
| 0.00 | 37.4 | 10 | 0 |
| 0.11 | 40.9 | 18 | 2 |
| 0.22 | 42.8 | 25 | 4 |
| 0.33 | 44.2 | 30 | 6 |
| 0.44 | 45.5 | 34 | 8 |
| 0.56 | 46.7 | 39 | - |
| 0.67 | 47.8 | 43 | 11 |
| 0.78 | 48.8 | 47 | 13 |
| 0.89 | 49.7 | 50 | 15 |
| 1.00 | 50.6 | 53 | 17 |
| 1.11 | 51.4 | 56 | 19 |
| 1.22 | 52.2 | 59 | 21 |
| 1.33 | 53.0 | 61 | 23 |
| 1.44 | 53.7 | 64 | 25 |
| 1.56 | 54.5 | 66 | 27 |
| 1.67 | 55.3 | 69 | 29 |
| 1.78 | 55.9 | 71 | 31 |
| 1.89 | 56.6 | 73 | 33 |
| 2.00 | 57.3 | 74 | 35 |
| 2.11 | 57.9 | 76 | 37 |
| 2.22 | 58.5 | 78 | 39 |
| 2.33 | 59.2 | 80 | 41 |
| 2.44 | 59.8 | 81 | 44 |
| 2.56 | 60.4 | 83 | 46 |
| 2.67 | 61.0 | 84 | 48 |
| 2.78 | 61.6 | 85 | 50 |
| 2.89 | 62.1 | 87 | 52 |
| 3.00 | 62.7 | 88 | 55 |
| 3.11 | 63.2 | 89 | 57 |
| 3.22 | 63.8 | 90 | 59 |
| 3.33 | 64.3 | 91 | 62 |
| 3.44 | 64.8 | 92 | 64 |
| 3.56 | 65.4 | 93 | 66 |
| 3.67 | 65.9 | 94 | 69 |
| 3.78 | 66.4 | 95 | 71 |
| 3.89 | 66.9 | 96 | 74 |
| 4.00 | 67.4 | - | 76 |
| 4.11 | 67.9 | 97 | 78 |
| 4.22 | 68.3 | 98 | 81 |
| 4.33 | 68.8 | - | 83 |
| 4.44 | 69.3 | 99 | 86 |
| 4.56 | 69.8 | 100 | 89 |
| 4.67 | 70.3 | 100 | 91 |
| 4.78 | 70.7 | 100 | 94 |
| 4.89 | 71.2 | - | 96 |
| 5.00 | 71.6 | 100 | 99 |
| NB: RS = Raw Score; T = T-score; PR_n = Percentile Rank score general population; PR_cl = Percentile Rank score clinical sample. | | | |
| ^1^Formula for population all (ID:17) for RS->TRankit: y=37.4+20604.229*(1-exp(-exp(5.936e-01*(ln(x+.0001)-ln(240429.745))))); a Weib2 function. | | | |
| ^2^Formula for population all (ID:17) for RS->PR_n: y=10.2+106.838*(1-exp(-exp(8.403e-01*(ln(x+.0001)-ln(2.206))))); a Weib2 function. | | | |
| ^3^Formula for clinical all (ID:12) for RS->PR_cl: y=-180.9+3750.034/(1+exp(-9.251e-02*(x-32.212))); a sigm-L4 function. | | | |

**Supplementary Table 5a**

*Cross-Walk Table from Raw Scores to T- and PR-scores for Body Image Concern for females and males*

|  | female | | | male | | |
| --- | --- | --- | --- | --- | --- | --- |
| RS | T1^1^ | PR_n1^2^ | PR_cl1^3^ | T2^4^ | PR_n2^5^ | PR_cl2^6^ |
| 0.00 | 37.2 | 10 | 1 | 38.5 | 13 | 1 |
| 0.11 | 40.6 | 18 | 2 | 43.4 | 24 | - |
| 0.22 | 42.4 | 23 | 4 | 45.5 | 32 | - |
| 0.33 | 43.8 | 28 | 6 | 47.0 | 38 | - |
| 0.44 | 45.1 | 33 | 8 | 48.4 | 44 | 7 |
| 0.56 | 46.3 | 38 | - | 49.6 | 49 | - |
| 0.67 | 47.4 | 42 | 11 | 50.7 | 53 | - |
| 0.78 | 48.3 | 45 | 13 | 51.7 | 57 | - |
| 0.89 | 49.3 | 48 | 15 | 52.6 | - | - |
| 1.00 | 50.1 | 52 | 17 | 53.4 | 64 | 17 |
| 1.11 | 51.0 | 55 | 19 | 54.2 | 67 | - |
| 1.22 | 51.8 | 57 | 21 | 55.0 | 69 | - |
| 1.33 | 52.6 | 60 | 22 | 55.7 | 72 | 24 |
| 1.44 | 53.4 | 62 | 24 | 56.4 | 74 | - |
| 1.56 | 54.2 | 65 | 27 | 57.2 | 76 | 29 |
| 1.67 | 54.9 | 67 | 29 | 57.8 | - | 32 |
| 1.78 | 55.6 | 69 | 31 | 58.4 | 80 | - |
| 1.89 | 56.3 | 71 | 33 | 59.1 | 81 | 37 |
| 2.00 | 56.9 | 73 | 35 | 59.6 | 83 | - |
| 2.11 | 57.6 | 75 | 37 | 60.2 | 84 | 41 |
| 2.22 | 58.2 | 77 | 39 | 60.8 | 85 | 44 |
| 2.33 | 58.8 | 79 | 41 | 61.3 | - | - |
| 2.44 | 59.5 | 80 | 43 | 61.9 | - | - |
| 2.56 | 60.1 | 82 | 45 | 62.4 | - | - |
| 2.67 | 60.7 | 83 | 47 | 63.0 | - | - |
| 2.78 | 61.3 | 85 | 50 | 63.5 | 90 | - |
| 2.89 | 61.9 | 86 | 52 | 64.0 | 91 | - |
| 3.00 | 62.4 | 87 | 54 | 64.4 | - | - |
| 3.11 | 63.0 | 88 | 56 | 64.9 | - | - |
| 3.22 | 63.5 | 90 | 59 | 65.4 | - | 66 |
| 3.33 | 64.1 | 91 | 61 | 65.8 | 94 | 68 |
| 3.44 | 64.6 | 92 | 63 | 66.3 | 94 | 70 |
| 3.56 | 65.2 | 93 | 66 | 66.8 | - | - |
| 3.67 | 65.7 | 94 | 68 | 67.2 | 95 | - |
| 3.78 | 66.2 | 95 | 71 | 67.6 | - | - |
| 3.89 | 66.7 | - | 73 | 68.1 | 96 | 78 |
| 4.00 | 67.3 | - | 76 | 68.5 | - | 80 |
| 4.11 | 67.8 | 97 | 78 | 68.9 | - | - |
| 4.22 | 68.2 | 98 | 81 | 69.3 | - | - |
| 4.33 | 68.7 | - | 83 | 69.7 | - | - |
| 4.44 | 69.2 | 100 | 86 | 70.1 | 98 | - |
| 4.56 | 69.7 | 100 | 89 | 70.5 | - | - |
| 4.67 | 70.2 | 100 | 91 | 70.9 | 98 | - |
| 4.78 | 70.7 | 100 | 94 | 71.3 | - | 92 |
| 4.89 | 71.2 | - | 96 | 71.7 | - | - |
| 5.00 | 71.6 | 100 | 99 | 72.1 | - | 95 |
| NB: RS = Raw Score; T = T-score; PR_n = Percentile Rank score general population; PR_cl = Percentile Rank score clinical sample. | | | | | | |
| ^1^Formula for population female (ID:17) for RS->TRankit: y=37.2+23653.503*(1-exp(-exp(6.081e-01*(ln(x+.0001)-ln(231406.594))))); a Weib2 function. | | | | | | |
| ^2^Formula for population female (ID:17) for RS->PR_n: y=9.8+109.496*(1-exp(-exp(8.523e-01*(ln(x+.0001)-ln(2.364))))); a Weib2 function. | | | | | | |
| ^3^Formula for clinical female (ID:12) for RS->PR_cl: y=-149.9+4525.703/(1+exp(-1.051e-01*(x-32.048))); a sigm-L4 function. | | | | | | |
| ^4^Formula for population male (ID:17) for RS->TRankit: y=38.5+3913.042*(1-exp(-exp(5.064e-01*(ln(x+.0001)-ln(59989.400))))); a Weib2 function. | | | | | | |
| ^5^Formula for population male (ID:17) for RS->PR_n: y=12.8+89.849*(1-exp(-exp(8.401e-01*(ln(x+.0001)-ln(1.228))))); a Weib2 function. | | | | | | |
| ^6^Formula for clinical male (ID:13) for RS->PR_cl: y=-10.4+139.852/((1+exp(-4.399e-01*(x+9.001)))^132.183); a sigm-L5 function. | | | | | | |

**Supplementary Table 6**

*Cross-Walk Table from Raw Scores to T- and PR-scores for Avoidance*

| RS | T^1^ | PR_n^2^ | PR_cl^3^ |
| --- | --- | --- | --- |
| 0.00 | 40.6 | 17 | 4 |
| 0.17 | 46.2 | 39 | 9 |
| 0.33 | 48.6 | 47 | 12 |
| 0.50 | 50.5 | 54 | 17 |
| 0.67 | 52.2 | 59 | 21 |
| 0.83 | 53.5 | 64 | 25 |
| 1.00 | 54.9 | 68 | 29 |
| 1.17 | 56.1 | 71 | 33 |
| 1.33 | 57.2 | 74 | 37 |
| 1.50 | 58.3 | 77 | 41 |
| 1.67 | 59.3 | 79 | 45 |
| 1.83 | 60.2 | 82 | 49 |
| 2.00 | 61.2 | 84 | 53 |
| 2.17 | 62.1 | 86 | 57 |
| 2.33 | 62.9 | 88 | 61 |
| 2.50 | 63.7 | 89 | 65 |
| 2.67 | 64.5 | 91 | 69 |
| 2.83 | 65.3 | 93 | 72 |
| 3.00 | 66.1 | 94 | 76 |
| 3.17 | 66.8 | 95 | 79 |
| 3.33 | 67.5 | 97 | 82 |
| 3.50 | 68.2 | 98 | 85 |
| 3.67 | 68.9 | 99 | 88 |
| 3.83 | 69.6 | - | 91 |
| 4.00 | 70.2 | 100 | 93 |
| 4.17 | 70.9 | 100 | 95 |
| 4.33 | 71.5 | - | 97 |
| 4.50 | 72.1 | 100 | 98 |
| 4.67 | 72.7 | 100 | 100 |
| 4.83 | 73.3 | - | 100 |
| 5.00 | 73.9 | - | 100 |
| NB: RS = Raw Score; T = T-score; PR_n = Percentile Rank score general population; PR_cl = Percentile Rank score clinical sample. | | | |
| ^1^Formula for population all (ID:17) for RS->TRankit: y=40.6+19155.007*(1-exp(-exp(5.280e-01*(ln(x+.0001)-ln(842324.602))))); a Weib2 function. | | | |
| ^2^Formula for population all (ID:17) for RS->PR_n: y=17.0+126.398*(1-exp(-exp(5.551e-01*(ln(x+.0001)-ln(3.342))))); a Weib2 function. | | | |
| ^3^Formula for clinical all (ID:13) for RS->PR_cl: y=-1800.6+1905.038/((1+exp(-1.577*(x-4.018)))^8.508e-03); a sigm-L5 function. | | | |

**Supplementary Table 6a**

*Cross-Walk Table from Raw Scores to T- and PR-scores for Avoidance for females and males*

|  | female | | | male | | |
| --- | --- | --- | --- | --- | --- | --- |
| RS | T1^1^ | PR_n1^2^ | PR_cl1^3^ | T2^4^ | PR_n2^5^ | PR_cl2^6^ |
| 0.00 | 40.4 | 16 | 5 | 42.3 | 22 | 7 |
| 0.17 | 45.7 | 37 | 9 | 50.2 | 52 | - |
| 0.33 | 48.0 | 45 | 12 | 52.7 | 62 | - |
| 0.50 | 49.9 | 52 | 16 | 54.7 | 68 | 16 |
| 0.67 | 51.6 | 57 | 20 | 56.2 | 73 | 21 |
| 0.83 | 53.0 | 62 | 24 | 57.5 | 77 | - |
| 1.00 | 54.3 | 66 | 28 | 58.8 | 80 | - |
| 1.17 | 55.6 | 69 | 32 | 59.9 | 83 | 38 |
| 1.33 | 56.7 | 72 | 36 | 60.8 | - | 44 |
| 1.50 | 57.8 | 75 | - | 61.8 | 87 | 51 |
| 1.67 | 58.8 | 78 | 44 | 62.6 | 88 | 58 |
| 1.83 | 59.8 | 80 | 47 | 63.4 | - | - |
| 2.00 | 60.8 | 83 | 51 | 64.2 | 91 | 68 |
| 2.17 | 61.7 | 85 | 55 | 65.0 | - | - |
| 2.33 | 62.5 | 87 | 59 | 65.6 | 93 | - |
| 2.50 | 63.4 | 89 | 63 | 66.3 | - | - |
| 2.67 | 64.2 | 90 | 67 | 67.0 | - | - |
| 2.83 | 65.0 | 92 | 71 | 67.6 | - | 83 |
| 3.00 | 65.8 | 94 | 75 | 68.2 | - | - |
| 3.17 | 66.6 | 95 | 79 | 68.8 | - | 86 |
| 3.33 | 67.3 | 97 | 82 | 69.4 | 98 | - |
| 3.50 | 68.0 | 98 | 86 | 69.9 | - | - |
| 3.67 | 68.8 | 99 | 90 | 70.5 | 99 | - |
| 3.83 | 69.4 | - | 92 | 71.0 | - | - |
| 4.00 | 70.1 | 100 | 95 | 71.5 | - | - |
| 4.17 | 70.8 | 100 | - | 72.0 | - | 88 |
| 4.33 | 71.5 | - | 98 | 72.4 | - | 89 |
| 4.50 | 72.1 | 100 | 98 | 72.9 | - | - |
| 4.67 | 72.8 | 100 | 99 | 73.4 | - | - |
| 4.83 | 73.4 | - | 99 | 73.8 | - | - |
| 5.00 | 74.0 | - | 99 | 74.3 | - | - |
| NB: RS = Raw Score; T = T-score; PR_n = Percentile Rank score general population; PR_cl = Percentile Rank score clinical sample. | | | | | | |
| ^1^Formula for population female (ID:17) for RS->TRankit: y=40.4+22383.670*(1-exp(-exp(5.464e-01*(ln(x+.0001)-ln(732483.140))))); a Weib2 function. | | | | | | |
| ^2^Formula for population female (ID:17) for RS->PR_n: y=16.2+133.088*(1-exp(-exp(5.702e-01*(ln(x+.0001)-ln(3.810))))); a Weib2 function. | | | | | | |
| ^3^Formula for clinical female (ID:13) for RS->PR_cl: y=-2771.3+2870.407/((1+exp(-4.060*(x-4.016)))^2.048e-03); a sigm-L5 function. | | | | | | |
| ^4^Formula for population male (ID:17) for RS->TRankit: y=42.3+3431.187*(1-exp(-exp(4.154e-01*(ln(x+.0001)-ln(382967.784))))); a Weib2 function. | | | | | | |
| ^5^Formula for population male (ID:17) for RS->PR_n: y=22.1+85.693*(1-exp(-exp(5.381e-01*(ln(x+.0001)-ln(8.037e-01))))); a Weib2 function. | | | | | | |
| ^6^Formula for clinical male (ID:11) for RS->PR_cl: y=89.022/(1+exp(-1.799*(x-1.335))); a sigm-L3 function. | | | | | | |

**Supplementary Table 7**

*Cross-Walk Table from Raw Scores to T- and PR-scores for Compulsive self-monitoring*

| RS | T^1^ | PR_n^2^ | PR_cl^3^ |
| --- | --- | --- | --- |
| 0.0 | 36.6 | 9 | 2 |
| 0.2 | 41.4 | 20 | 6 |
| 0.4 | 44.0 | 29 | 9 |
| 0.6 | 46.2 | 37 | 12 |
| 0.8 | 48.2 | 44 | 16 |
| 1.0 | 50.0 | 51 | 20 |
| 1.2 | 51.7 | 57 | 24 |
| 1.4 | 53.2 | 62 | 28 |
| 1.6 | 54.7 | 67 | 32 |
| 1.8 | 56.1 | 71 | 37 |
| 2.0 | 57.5 | 75 | 41 |
| 2.2 | 58.8 | 79 | 45 |
| 2.4 | 60.1 | 82 | 50 |
| 2.6 | 61.4 | 85 | 54 |
| 2.8 | 62.6 | 88 | 58 |
| 3.0 | 63.8 | 90 | 63 |
| 3.2 | 64.9 | 92 | 67 |
| 3.4 | 66.0 | 94 | 71 |
| 3.6 | 67.1 | 96 | 75 |
| 3.8 | 68.2 | 98 | 80 |
| 4.0 | 69.3 | 99 | 84 |
| 4.2 | 70.3 | 100 | 87 |
| 4.4 | 71.4 | 100 | 91 |
| 4.6 | 72.4 | - | 95 |
| 4.8 | 73.4 | 100 | 99 |
| 5.0 | 74.4 | 100 | 100 |
| NB: RS = Raw Score; T = T-score; PR_n = Percentile Rank score general population; PR_cl = Percentile Rank score clinical sample. | | | |
| ^1^Formula for population all (ID:17) for RS->TRankit: y=36.6+31516.907*(1-exp(-exp(6.451e-01*(ln(x+.0001)-ln(168926.465))))); a Weib2 function. | | | |
| ^2^Formula for population all (ID:17) for RS->PR_n: y=8.9+107.490*(1-exp(-exp(9.499e-01*(ln(x+.0001)-ln(2.094))))); a Weib2 function. | | | |
| ^3^Formula for clinical all (ID:15) for RS->PR_cl: y=-21.1+192.914*(exp(-exp(-3.092e-01*(x-2.410)))); a gompertz2 function. | | | |

**Supplementary Table 7a**

*Cross-Walk Table from Raw Scores to T- and PR-scores for Compulsive self-monitoring for females and males*

|  | female | | | male | | |
| --- | --- | --- | --- | --- | --- | --- |
| RS | T1^1^ | PR_n1^2^ | PR_cl1^3^ | T2^4^ | PR_n2^5^ | PR_cl2^6^ |
| 0.0 | 36.2 | 8 | 2 | 39.0 | 13 | 6 |
| 0.2 | 40.8 | 18 | 5 | 45.2 | 34 | 11 |
| 0.4 | 43.4 | 27 | 8 | 48.2 | 45 | 16 |
| 0.6 | 45.6 | 35 | 12 | 50.6 | 54 | - |
| 0.8 | 47.6 | 42 | 15 | 52.6 | 60 | - |
| 1.0 | 49.4 | 49 | 19 | 54.5 | 66 | 31 |
| 1.2 | 51.0 | 55 | 22 | 56.1 | 71 | 36 |
| 1.4 | 52.6 | 60 | 26 | 57.7 | 76 | - |
| 1.6 | 54.1 | 65 | 30 | 59.2 | 80 | 47 |
| 1.8 | 55.6 | 70 | 34 | 60.6 | 83 | - |
| 2.0 | 57.0 | 74 | 39 | 61.9 | 86 | 58 |
| 2.2 | 58.3 | 77 | 43 | 63.2 | 89 | 63 |
| 2.4 | 59.6 | 81 | 47 | 64.4 | 91 | 69 |
| 2.6 | 60.9 | 84 | 52 | 65.6 | - | 75 |
| 2.8 | 62.1 | 87 | 56 | 66.7 | 95 | 80 |
| 3.0 | 63.3 | 89 | 61 | 67.8 | 97 | - |
| 3.2 | 64.5 | 92 | 65 | 68.9 | - | - |
| 3.4 | 65.6 | 94 | 70 | 69.9 | - | - |
| 3.6 | 66.8 | 96 | 74 | 70.9 | - | - |
| 3.8 | 67.9 | 97 | 79 | 71.9 | 100 | - |
| 4.0 | 69.0 | 99 | 83 | 72.9 | - | - |
| 4.2 | 70.0 | 100 | 87 | 73.8 | - | - |
| 4.4 | 71.1 | 100 | 91 | 74.8 | - | 92 |
| 4.6 | 72.1 | - | 95 | 75.7 | - | 92 |
| 4.8 | 73.1 | 100 | 99 | 76.6 | - | - |
| 5.0 | 74.1 | 100 | 100 | 77.5 | - | - |
| NB: RS = Raw Score; T = T-score; PR_n = Percentile Rank score general population; PR_cl = Percentile Rank score clinical sample. | | | | | | |
| ^1^Formula for population female (ID:17) for RS->TRankit: y=36.2+28737.691*(1-exp(-exp(6.574e-01*(ln(x+.0001)-ln(119831.427))))); a Weib2 function. | | | | | | |
| ^2^Formula for population female (ID:17) for RS->PR_n: y=8.3+106.990*(1-exp(-exp(9.974e-01*(ln(x+.0001)-ln(2.122))))); a Weib2 function. | | | | | | |
| ^3^Formula for clinical female (ID:12) for RS->PR_cl: y=-38.9+199.780/(1+exp(-4.513e-01*(x-3.004))); a sigm-L4 function. | | | | | | |
| ^4^Formula for population male (ID:17) for RS->TRankit: y=39.0+16458.351*(1-exp(-exp(5.680e-01*(ln(x+.0001)-ln(214308.522))))); a Weib2 function. | | | | | | |
| ^5^Formula for population male (ID:17) for RS->PR_n: y=13.3+107.160*(1-exp(-exp(7.314e-01*(ln(x+.0001)-ln(1.683))))); a Weib2 function. | | | | | | |
| ^6^Formula for clinical male (ID:13) for RS->PR_cl: y=-388.2+479.912/((1+exp(-16.135*(x-3.188)))^3.839e-03); a sigm-L5 function. | | | | | | |

**Supplementary Table 8**

*Cross-Walk Table from Raw Scores to T- and PR-scores for Depersonalization*

| RS | T^1^ | PR_n^2^ | PR_cl^3^ |
| --- | --- | --- | --- |
| 0.00 | 40.0 | 15 | 4 |
| 0.17 | 45.7 | 36 | 7 |
| 0.33 | 48.0 | 45 | 10 |
| 0.50 | 50.0 | 52 | 13 |
| 0.67 | 51.6 | 57 | 17 |
| 0.83 | 53.0 | 62 | 20 |
| 1.00 | 54.3 | 66 | 23 |
| 1.17 | 55.5 | 70 | 27 |
| 1.33 | 56.6 | 73 | 30 |
| 1.50 | 57.6 | 76 | 34 |
| 1.67 | 58.6 | 78 | 38 |
| 1.83 | 59.5 | 81 | 41 |
| 2.00 | 60.5 | 83 | 45 |
| 2.17 | 61.4 | 85 | 49 |
| 2.33 | 62.2 | 86 | 53 |
| 2.50 | 63.0 | 88 | 57 |
| 2.67 | 63.8 | 90 | 61 |
| 2.83 | 64.5 | 91 | 65 |
| 3.00 | 65.3 | 93 | 69 |
| 3.17 | 66.0 | 94 | 73 |
| 3.33 | 66.7 | 95 | 76 |
| 3.50 | 67.4 | 96 | 80 |
| 3.67 | 68.1 | 97 | 83 |
| 3.83 | 68.7 | 98 | 86 |
| 4.00 | 69.3 | 99 | 89 |
| 4.17 | 70.0 | 100 | 92 |
| 4.33 | 70.6 | 100 | 94 |
| 4.50 | 71.2 | - | 96 |
| 4.67 | 71.8 | 100 | 97 |
| 4.83 | 72.4 | - | 98 |
| 5.00 | 72.9 | 100 | 99 |
| NB: RS = Raw Score; T = T-score; PR_n = Percentile Rank score general population; PR_cl = Percentile Rank score clinical sample. | | | |
| ^1^Formula for population all (ID:17) for RS->TRankit: y=40.0+14775.243*(1-exp(-exp(5.199e-01*(ln(x+.0001)-ln(628972.853))))); a Weib2 function. | | | |
| ^2^Formula for population all (ID:17) for RS->PR_n: y=15.5+104.211*(1-exp(-exp(6.392e-01*(ln(x+.0001)-ln(1.890))))); a Weib2 function. | | | |
| ^3^Formula for clinical all (ID:13) for RS->PR_cl: y=-129.3+232.164/((1+exp(-1.701*(x-4.080)))^7.992e-02); a sigm-L5 function. | | | |

**Supplementary Table 8a**

*Cross-Walk Table from Raw Scores to T- and PR-scores for Depersonalization for females and males*

|  | female | | | male | | |
| --- | --- | --- | --- | --- | --- | --- |
| RS | T1^1^ | PR_n1^2^ | PR_cl1^3^ | T2^4^ | PR_n2^5^ | PR_cl2^6^ |
| 0.00 | 39.6 | 15 | 3 | 42.3 | 22 | 0 |
| 0.17 | 45.2 | 33 | 6 | 49.6 | 49 | 15 |
| 0.33 | 47.5 | 42 | 9 | 51.9 | 60 | 21 |
| 0.50 | 49.4 | 50 | 12 | 53.8 | 66 | 27 |
| 0.67 | 51.1 | 56 | 15 | 55.3 | 71 | - |
| 0.83 | 52.5 | 60 | 18 | 56.6 | 74 | - |
| 1.00 | 53.8 | 65 | 22 | 57.8 | 77 | 41 |
| 1.17 | 55.0 | 68 | 25 | 58.8 | - | - |
| 1.33 | 56.1 | 72 | 28 | 59.8 | 82 | - |
| 1.50 | 57.2 | 75 | 32 | 60.7 | - | 52 |
| 1.67 | 58.2 | 77 | 36 | 61.5 | 86 | - |
| 1.83 | 59.2 | 80 | 39 | 62.3 | - | 58 |
| 2.00 | 60.1 | 82 | 43 | 63.1 | 88 | - |
| 2.17 | 61.0 | 84 | 47 | 63.8 | 90 | 64 |
| 2.33 | 61.8 | 86 | 51 | 64.5 | 91 | 66 |
| 2.50 | 62.7 | 88 | 55 | 65.1 | - | - |
| 2.67 | 63.5 | 89 | 60 | 65.8 | 93 | 72 |
| 2.83 | 64.3 | 91 | 64 | 66.4 | 94 | - |
| 3.00 | 65.0 | 92 | 68 | 67.0 | - | - |
| 3.17 | 65.8 | 93 | 72 | 67.6 | - | 79 |
| 3.33 | 66.5 | 95 | 76 | 68.1 | - | - |
| 3.50 | 67.2 | 96 | 79 | 68.7 | - | 84 |
| 3.67 | 67.9 | 97 | 83 | 69.2 | - | - |
| 3.83 | 68.5 | 98 | 86 | 69.7 | - | - |
| 4.00 | 69.2 | 99 | 89 | 70.2 | - | - |
| 4.17 | 69.9 | 100 | 91 | 70.7 | - | 93 |
| 4.33 | 70.5 | 100 | 94 | 71.2 | 100 | - |
| 4.50 | 71.1 | - | 96 | 71.6 | - | - |
| 4.67 | 71.7 | 100 | 97 | 72.1 | - | 99 |
| 4.83 | 72.3 | - | 98 | 72.5 | - | - |
| 5.00 | 72.9 | 100 | 100 | 73.0 | - | - |
| NB: RS = Raw Score; T = T-score; PR_n = Percentile Rank score general population; PR_cl = Percentile Rank score clinical sample. | | | | | | |
| ^1^Formula for population female (ID:17) for RS->TRankit: y=39.6+15537.703*(1-exp(-exp(5.304e-01*(ln(x+.0001)-ln(536039.504))))); a Weib2 function. | | | | | | |
| ^2^Formula for population female (ID:17) for RS->PR_n: y=14.5+104.965*(1-exp(-exp(6.653e-01*(ln(x+.0001)-ln(1.923))))); a Weib2 function. | | | | | | |
| ^3^Formula for clinical female (ID:13) for RS->PR_cl: y=-74.6+178.013/((1+exp(-1.597*(x-3.949)))^1.307e-01); a sigm-L5 function. | | | | | | |
| ^4^Formula for population male (ID:17) for RS->TRankit: y=42.3+4579.070*(1-exp(-exp(4.279e-01*(ln(x+.0001)-ln(598833.702))))); a Weib2 function. | | | | | | |
| ^5^Formula for population male (ID:18) for RS->PR_n: y=678.1-656.007/((1+(x/3.872e-02)^1.421)^1.902e-02); a logis5 function. | | | | | | |
| ^6^Formula for clinical male (ID:7) for RS->PR_cl: y=(x^5.792e-01)/2.456e-02; a power2 function. | | | | | | |

**Supplementary Table 9**

*Cross-Walk Table from Raw Scores to T- and PR-scores for BUT-B Positive Symptom Distress Index*

| RS | T^1^ | PR_n^2^ | PR_cl^3^ |
| --- | --- | --- | --- |
| 0.00 | 37.3 | 10 | 2 |
| 0.03 | 40.6 | 18 | 3 |
| 0.05 | 41.6 | 21 | - |
| 0.08 | 42.7 | 25 | 5 |
| 0.11 | 43.6 | 28 | 6 |
| 0.14 | 44.3 | 31 | 7 |
| 0.16 | 44.8 | 32 | 8 |
| 0.19 | 45.4 | 35 | 9 |
| 0.22 | 46.0 | 37 | 10 |
| 0.24 | 46.4 | 38 | - |
| 0.27 | 47.0 | 40 | 12 |
| 0.30 | 47.5 | 42 | - |
| 0.32 | 47.8 | 43 | 14 |
| 0.35 | 48.2 | 45 | 15 |
| 0.38 | 48.7 | 46 | 16 |
| 0.41 | 49.1 | 48 | - |
| 0.43 | 49.4 | 49 | - |
| 0.46 | 49.8 | 50 | 19 |
| 0.49 | 50.2 | 52 | 20 |
| 0.51 | 50.4 | 53 | 21 |
| 0.54 | 50.8 | 54 | 22 |
| 0.57 | 51.1 | 55 | 23 |
| 0.59 | 51.4 | 56 | 24 |
| 0.62 | 51.7 | 57 | 25 |
| 0.65 | 52.0 | 58 | 26 |
| 0.68 | 52.4 | 59 | 28 |
| 0.70 | 52.6 | 60 | 28 |
| 0.73 | 52.9 | 61 | - |
| 0.76 | 53.2 | 62 | 31 |
| 0.78 | 53.4 | 63 | 32 |
| 0.81 | 53.7 | 63 | - |
| 0.84 | 54.0 | 64 | 34 |
| 0.86 | 54.2 | 65 | - |
| 0.89 | 54.4 | 66 | - |
| 0.92 | 54.7 | 67 | 37 |
| 0.95 | 55.0 | 67 | 38 |
| 0.97 | 55.2 | 68 | 39 |
| 1.00 | 55.4 | 69 | 40 |
| 1.03 | 55.7 | 70 | - |
| 1.05 | 55.9 | 70 | - |
| 1.08 | 56.1 | 71 | 43 |
| 1.11 | 56.4 | 72 | 44 |
| 1.14 | 56.6 | 72 | - |
| 1.16 | 56.8 | 73 | 46 |
| 1.19 | 57.0 | 73 | 47 |
| 1.22 | 57.2 | 74 | 48 |
| 1.24 | 57.4 | 75 | 49 |
| 1.27 | 57.6 | 75 | 50 |
| 1.30 | 57.9 | 76 | 51 |
| 1.32 | 58.0 | 76 | 52 |
| 1.35 | 58.2 | 77 | 53 |
| 1.38 | 58.5 | 77 | 54 |
| 1.41 | 58.7 | 78 | 55 |
| 1.43 | 58.8 | 78 | 56 |
| 1.46 | 59.0 | 79 | 57 |
| 1.49 | 59.3 | 79 | 58 |
| 1.51 | 59.4 | 80 | 58 |
| 1.54 | 59.6 | 80 | - |
| 1.57 | 59.8 | - | - |
| 1.59 | 59.9 | - | 61 |
| 1.62 | 60.2 | 82 | 62 |
| 1.65 | 60.4 | 82 | 63 |
| 1.68 | 60.6 | 83 | 64 |
| 1.70 | 60.7 | 83 | 64 |
| 1.73 | 60.9 | 83 | 65 |
| 1.76 | 61.1 | 84 | 66 |
| 1.78 | 61.2 | 84 | 66 |
| 1.81 | 61.4 | 85 | 67 |
| 1.84 | 61.6 | 85 | 68 |
| 1.86 | 61.7 | 85 | 69 |
| 1.89 | 61.9 | 86 | 70 |
| 1.92 | 62.1 | 86 | - |
| 1.95 | 62.3 | 87 | - |
| 1.97 | 62.4 | - | 72 |
| 2.00 | 62.6 | 87 | - |
| 2.03 | 62.8 | 88 | - |
| 2.05 | 62.9 | 88 | - |
| 2.08 | 63.1 | 88 | - |
| 2.11 | 63.2 | 89 | 75 |
| 2.14 | 63.4 | 89 | 76 |
| 2.16 | 63.5 | 89 | 76 |
| 2.19 | 63.7 | 90 | 77 |
| 2.22 | 63.9 | - | - |
| 2.24 | 64.0 | 90 | - |
| 2.27 | 64.2 | - | 79 |
| 2.30 | 64.3 | 91 | - |
| 2.32 | 64.5 | 91 | - |
| 2.35 | 64.6 | - | 80 |
| 2.38 | 64.8 | 92 | 81 |
| 2.41 | 65.0 | 92 | - |
| 2.43 | 65.1 | 92 | 82 |
| 2.46 | 65.2 | 93 | 82 |
| 2.49 | 65.4 | 93 | - |
| 2.51 | 65.5 | 93 | 83 |
| 2.54 | 65.7 | 94 | 84 |
| 2.57 | 65.8 | - | 84 |
| 2.59 | 65.9 | 94 | 85 |
| 2.62 | 66.1 | 94 | 85 |
| 2.65 | 66.2 | 95 | 85 |
| 2.68 | 66.4 | 95 | - |
| 2.70 | 66.5 | 95 | 86 |
| 2.73 | 66.7 | - | - |
| 2.76 | 66.8 | 96 | - |
| 2.78 | 66.9 | - | 87 |
| 2.81 | 67.1 | - | - |
| 2.84 | 67.2 | 96 | - |
| 2.86 | 67.3 | - | - |
| 2.89 | 67.5 | 97 | - |
| 2.92 | 67.6 | 97 | 89 |
| 2.95 | 67.8 | 97 | - |
| 2.97 | 67.9 | - | - |
| 3.00 | 68.0 | - | 90 |
| 3.03 | 68.2 | 98 | - |
| 3.05 | 68.3 | - | - |
| 3.08 | 68.4 | - | 91 |
| 3.11 | 68.6 | 99 | - |
| 3.14 | 68.7 | - | - |
| 3.16 | 68.8 | 99 | - |
| 3.19 | 68.9 | 99 | - |
| 3.22 | 69.1 | - | - |
| 3.24 | 69.2 | 100 | - |
| 3.27 | 69.3 | - | 93 |
| 3.30 | 69.5 | - | 93 |
| 3.32 | 69.6 | - | - |
| 3.35 | 69.7 | - | - |
| 3.38 | 69.8 | - | - |
| 3.41 | 70.0 | 100 | 94 |
| 3.43 | 70.1 | - | - |
| 3.46 | 70.2 | - | 94 |
| 3.49 | 70.3 | - | 95 |
| 3.51 | 70.4 | - | 95 |
| 3.54 | 70.6 | - | 95 |
| 3.57 | 70.7 | 100 | 95 |
| 3.59 | 70.8 | - | - |
| 3.62 | 70.9 | - | - |
| 3.65 | 71.1 | - | - |
| 3.68 | 71.2 | - | - |
| 3.70 | 71.3 | - | - |
| 3.73 | 71.4 | - | - |
| 3.76 | 71.5 | - | - |
| 3.78 | 71.6 | - | - |
| 3.81 | 71.8 | - | - |
| 3.84 | 71.9 | - | - |
| 3.86 | 72.0 | - | - |
| 3.89 | 72.1 | - | - |
| 3.92 | 72.2 | - | - |
| 3.95 | 72.4 | - | 97 |
| 3.97 | 72.4 | - | 98 |
| 4.00 | 72.6 | - | - |
| 4.03 | 72.7 | - | - |
| 4.05 | 72.8 | - | - |
| 4.08 | 72.9 | - | - |
| 4.11 | 73.0 | 100 | - |
| 4.14 | 73.2 | - | - |
| 4.16 | 73.2 | - | 98 |
| 4.19 | 73.4 | - | - |
| 4.22 | 73.5 | 100 | - |
| 4.24 | 73.6 | - | 99 |
| 4.27 | 73.7 | - | - |
| 4.30 | 73.8 | - | - |
| 4.32 | 73.9 | - | - |
| 4.35 | 74.0 | - | - |
| 4.38 | 74.1 | - | - |
| 4.41 | 74.3 | 100 | - |
| 4.43 | 74.3 | - | - |
| 4.46 | 74.5 | - | - |
| 4.49 | 74.6 | - | - |
| 4.51 | 74.7 | - | - |
| 4.54 | 74.8 | - | - |
| 4.57 | 74.9 | - | - |
| 4.59 | 75.0 | - | - |
| 4.62 | 75.1 | - | - |
| 4.65 | 75.2 | - | - |
| 4.68 | 75.3 | - | - |
| 4.70 | 75.4 | - | - |
| 4.73 | 75.5 | - | - |
| 4.76 | 75.6 | - | - |
| 4.78 | 75.7 | - | - |
| 4.81 | 75.8 | - | - |
| 4.84 | 76.0 | - | - |
| 4.86 | 76.0 | - | - |
| 4.89 | 76.1 | - | - |
| 4.92 | 76.3 | - | - |
| 4.95 | 76.4 | - | - |
| 4.97 | 76.4 | - | - |
| 5.00 | 76.6 | - | - |
| NB: RS = Raw Score; T = T-score; PR_n = Percentile Rank score general population; PR_cl = Percentile Rank score clinical sample. | | | |
| ^1^Formula for population all (ID:17) for RS->TRankit: y=37.3+12439.611*(1-exp(-exp(4.800e-01*(ln(x+.0001)-ln(807004.900))))); a Weib2 function. | | | |
| ^2^Formula for population all (ID:17) for RS->PR_n: y=9.8+111.935*(1-exp(-exp(6.561e-01*(ln(x+.0001)-ln(1.553))))); a Weib2 function. | | | |
| ^3^Formula for clinical all (ID:12) for RS->PR_cl: y=-46.7+148.743/(1+exp(-1.055*(x-6.810e-01))); a sigm-L4 function. | | | |

**Supplementary Table 9a**

*Cross-Walk Table from Raw Scores to T- and PR-scores for BUT-B Positive Symptom Distress Index*

*for females and males*

|  | female | | | male | | |
| --- | --- | --- | --- | --- | --- | --- |
| RS | T1^1^ | PR_n1^2^ | PR_cl1^3^ | T2^4^ | PR_n2^5^ | PR_cl2^6^ |
| 0.00 | 37 | 9 | 2 | 40,8 | 17 | 6 |
| 0,03 | 40,2 | 16 | 3 | 43,9 | 30 | 6 |
| 0,05 | 41,1 | 19 | 4 | 44,8 | 34 | 6 |
| 0,08 | 42,2 | 23 | 4 | 45,9 | 38 | 7 |
| 0,11 | 43,1 | 26 | 5 | 46,8 | 41 | 9 |
| 0,14 | 43,9 | 29 | 6 | 47,6 | 44 | 11 |
| 0,16 | 44,4 | 31 | 7 | 48 | 45 | 13 |
| 0,19 | 45.0 | 33 | 8 | 48,6 | 48 | 17 |
| 0,22 | 45,6 | 35 | 8 | 49,2 | 50 | 20 |
| 0,24 | 46 | 37 | 9 | 49,6 | 51 | 22 |
| 0,27 | 46,5 | 38 | 10 | 50,1 | 52 | 26 |
| 0,30 | 47,1 | 40 | 11 | 50,6 | 54 | 29 |
| 0,32 | 47,4 | 42 | 12 | 50,9 | 55 | 31 |
| 0,35 | 47,8 | 43 | 12 | 51,4 | 56 | 34 |
| 0,38 | 48,3 | 45 | 13 | 51,8 | 58 | 37 |
| 0,41 | 48,7 | 46 | 14 | 52,2 | 59 | 39 |
| 0,43 | 49.0 | 47 | 15 | 52,5 | 60 | 41 |
| 0,46 | 49,4 | 49 | 16 | 52,9 | 61 | 43 |
| 0,49 | 49,8 | 50 | 17 | 53,3 | 62 | 46 |
| 0,51 | 50.0 | 51 | 18 | 53,5 | 63 | 47 |
| 0,54 | 50,4 | 52 | 19 | 53,9 | 64 | 49 |
| 0,57 | 50,8 | 54 | 20 | 54,2 | 65 | 51 |
| 0,59 | 51 | 54 | 21 | 54,4 | 66 | 52 |
| 0,62 | 51,3 | 56 | 22 | 54,8 | 67 | 54 |
| 0,65 | 51,7 | 57 | 23 | 55,1 | 68 | 55 |
| 0,68 | 52.0 | 58 | 24 | 55,4 | 68 | 57 |
| 0,70 | 52,2 | 58 | 25 | 55,6 | 69 | 57 |
| 0,73 | 52,5 | 60 | 26 | 55,9 | 70 | 59 |
| 0,76 | 52,8 | 61 | 27 | 56,2 | 71 | 60 |
| 0,78 | 53.0 | 61 | 28 | 56,4 | 71 | 61 |
| 0,81 | 53,3 | 62 | 29 | 56,7 | 72 | 62 |
| 0,84 | 53,6 | 63 | 30 | 57 | 73 | 63 |
| 0,86 | 53,8 | 64 | 31 | 57,2 | 73 | 64 |
| 0,89 | 54,1 | 65 | 32 | 57,5 | 74 | 65 |
| 0,92 | 54,4 | 65 | 33 | 57,7 | 75 | 66 |
| 0,95 | 54,6 | 66 | 34 | 58 | 75 | 67 |
| 0,97 | 54,8 | 67 | 35 | 58,2 | 76 | 67 |
| 1.00 | 55,1 | 68 | 36 | 58,4 | 77 | 68 |
| 1,03 | 55,3 | 68 | 37 | 58,7 | 77 | 69 |
| 1,05 | 55,5 | 69 | 38 | 58,8 | 78 | 69 |
| 1,08 | 55,8 | 70 | 39 | 59,1 | 78 | 70 |
| 1,11 | 56.0 | 70 | 41 | 59,3 | 79 | 71 |
| 1,14 | 56,3 | 71 | 42 | 59,6 | 80 | 71 |
| 1,16 | 56,4 | 72 | 43 | 59,7 | 80 | 72 |
| 1,19 | 56,7 | 72 | 44 | 60 | 81 | 73 |
| 1,22 | 56,9 | 73 | 45 | 60,2 | 81 | 73 |
| 1,24 | 57,1 | 74 | 46 | 60,3 | 81 | 74 |
| 1,27 | 57,3 | 74 | 47 | 60,6 | 82 | 74 |
| 1,30 | 57,5 | 75 | 48 | 60,8 | 83 | 75 |
| 1,32 | 57,7 | 75 | 49 | 60,9 | 83 | 75 |
| 1,35 | 57,9 | 76 | 50 | 61,2 | 83 | 76 |
| 1,38 | 58,1 | 76 | 51 | 61,4 | 84 | 76 |
| 1,41 | 58,4 | 77 | 52 | 61,6 | 85 | 77 |
| 1,43 | 58,5 | 77 | 53 | 61,7 | 85 | 77 |
| 1,46 | 58,7 | 78 | 54 | 61,9 | 85 | 78 |
| 1,49 | 58,9 | 79 | 55 | 62,1 | 86 | 78 |
| 1,51 | 59,1 | 79 | 56 | 62,3 | 86 | 78 |
| 1,54 | 59,3 | 79 | 57 | 62,5 | 87 | 79 |
| 1,57 | 59,5 | 80 | 58 | 62,7 | 87 | 79 |
| 1,59 | 59,6 | 80 | 59 | 62,8 | 87 | 79 |
| 1,62 | 59,8 | 81 | 60 | 63 | 88 | 80 |
| 1,65 | 60 | 81 | 61 | 63,2 | 88 | 80 |
| 1,68 | 60,2 | 82 | 62 | 63,4 | 89 | 81 |
| 1,70 | 60,4 | 82 | 62 | 63,5 | 89 | 81 |
| 1,73 | 60,6 | 83 | 63 | 63,7 | 89 | 81 |
| 1,76 | 60,8 | 83 | 64 | 63,9 | 90 | 82 |
| 1,78 | 60,9 | 83 | 65 | 64,1 | 90 | 82 |
| 1,81 | 61,1 | 84 | 66 | 64,2 | 91 | 82 |
| 1,84 | 61,3 | 84 | 67 | 64,4 | 91 | 83 |
| 1,86 | 61,4 | 85 | 67 | 64,5 | 91 | 83 |
| 1,89 | 61,6 | 85 | 68 | 64,7 | 92 | 83 |
| 1,92 | 61,8 | 86 | 69 | 64,9 | 92 | 83 |
| 1,95 | 62 | 86 | 70 | 65,1 | 92 | 84 |
| 1,97 | 62,1 | 86 | 71 | 65,2 | 93 | 84 |
| 2.00 | 62,3 | 87 | 72 | 65,4 | 93 | 84 |
| 2,03 | 62,5 | 87 | 72 | 65,6 | 94 | 85 |
| 2,05 | 62,6 | 87 | 73 | 65,7 | 94 | 85 |
| 2,08 | 62,8 | 88 | 74 | 65,9 | 94 | 85 |
| 2,11 | 63 | 88 | 74 | 66 | 94 | 85 |
| 2,14 | 63,1 | 89 | 75 | 66,2 | 95 | 86 |
| 2,16 | 63,2 | 89 | 76 | 66,3 | 95 | 86 |
| 2,19 | 63,4 | 89 | 76 | 66,5 | 95 | 86 |
| 2,22 | 63,6 | 90 | 77 | 66,7 | 96 | 86 |
| 2,24 | 63,7 | 90 | 78 | 66,8 | 96 | 86 |
| 2,27 | 63,9 | 90 | 78 | 66,9 | 96 | 87 |
| 2,30 | 64,1 | 90 | 79 | 67,1 | 97 | 87 |
| 2,32 | 64,2 | 91 | 79 | 67,2 | 97 | 87 |
| 2,35 | 64,3 | 91 | 80 | 67,4 | 97 | 87 |
| 2,38 | 64,5 | 91 | 81 | 67,5 | 98 | 88 |
| 2,41 | 64,7 | 92 | 81 | 67,7 | 98 | 88 |
| 2,43 | 64,8 | 92 | 82 | 67,8 | 98 | 88 |
| 2,46 | 64,9 | 92 | 82 | 68 | 98 | 88 |
| 2,49 | 65,1 | 93 | 83 | 68,1 | 99 | 88 |
| 2,51 | 65,2 | 93 | 83 | 68,2 | 99 | 89 |
| 2,54 | 65,4 | 93 | 84 | 68,4 | 99 | 89 |
| 2,57 | 65,5 | 93 | 84 | 68,5 | 100 | 89 |
| 2,59 | 65,6 | 94 | 85 | 68,6 | 100 | 89 |
| 2,62 | 65,8 | 94 | 85 | 68,8 | 100 | 89 |
| 2,65 | 66.0 | 94 | 86 | 68,9 | 100 | 90 |
| 2,68 | 66,1 | 94 | 86 | 69,1 | 100 | 90 |
| 2,70 | 66,2 | 95 | 86 | 69,2 | 100 | 90 |
| 2,73 | 66,4 | 95 | 87 | 69,3 | 100 | 90 |
| 2,76 | 66,5 | 95 | 87 | 69,5 | 100 | 90 |
| 2,78 | 66,6 | 95 | 87 | 69,6 | 100 | 90 |
| 2,81 | 66,8 | 96 | 88 | 69,7 | 100 | 91 |
| 2,84 | 66,9 | 96 | 88 | 69,9 | 100 | 91 |
| 2,86 | 67.0 | 96 | 89 | 70.0 | 100 | 91 |
| 2,89 | 67,2 | 96 | 89 | 70,1 | 100 | 91 |
| 2,92 | 67,4 | 97 | 89 | 70,3 | 100 | 91 |
| 2,95 | 67,5 | 97 | 90 | 70,4 | 100 | 91 |
| 2,97 | 67,6 | 97 | 90 | 70,5 | 100 | 91 |
| 3.00 | 67,7 | 97 | 90 | 70,7 | 100 | 92 |
| 3,03 | 67,9 | 97 | 91 | 70,8 | 100 | 92 |
| 3,05 | 68.0 | 98 | 91 | 70,9 | 100 | 92 |
| 3,08 | 68,1 | 98 | 91 | 71.0 | 100 | 92 |
| 3,11 | 68,3 | 98 | 91 | 71,2 | 100 | 92 |
| 3,14 | 68,4 | 98 | 92 | 71,3 | 100 | 92 |
| 3,16 | 68,5 | 99 | 92 | 71,4 | 100 | 92 |
| 3,19 | 68,7 | 99 | 92 | 71,6 | 100 | 93 |
| 3,22 | 68,8 | 99 | 92 | 71,7 | 100 | 93 |
| 3,24 | 68,9 | 99 | 93 | 71,8 | 100 | 93 |
| 3,27 | 69,1 | 99 | 93 | 71,9 | 100 | 93 |
| 3,30 | 69,2 | 100 | 93 | 72,1 | 100 | 93 |
| 3,32 | 69,3 | 100 | 93 | 72,2 | 100 | 93 |
| 3,35 | 69,4 | 100 | 93 | 72,3 | 100 | 93 |
| 3,38 | 69,6 | 100 | 94 | 72,4 | 100 | 93 |
| 3,41 | 69,7 | 100 | 94 | 72,6 | 100 | 94 |
| 3,43 | 69,8 | 100 | 94 | 72,6 | 100 | 94 |
| 3,46 | 69,9 | 100 | 94 | 72,8 | 100 | 94 |
| 3,49 | 70,1 | 100 | 94 | 72,9 | 100 | 94 |
| 3,51 | 70,2 | 100 | 94 | 73.0 | 100 | 94 |
| 3,54 | 70,3 | 100 | 95 | 73,1 | 100 | 94 |
| 3,57 | 70,4 | 100 | 95 | 73,3 | 100 | 94 |
| 3,59 | 70,5 | 100 | 95 | 73,3 | 100 | 94 |
| 3,62 | 70,7 | 100 | 95 | 73,5 | 100 | 95 |
| 3,65 | 70,8 | 100 | 95 | 73,6 | 100 | 95 |
| 3,68 | 70,9 | 100 | 95 | 73,7 | 100 | 95 |
| 3,70 | 71.0 | 100 | 96 | 73,8 | 100 | 95 |
| 3,73 | 71,1 | 100 | 96 | 73,9 | 100 | 95 |
| 3,76 | 71,3 | 100 | 96 | 74,1 | 100 | 95 |
| 3,78 | 71,4 | 100 | 96 | 74,2 | 100 | 95 |
| 3,81 | 71,5 | 100 | 96 | 74,3 | 100 | 95 |
| 3,84 | 71,6 | 100 | 96 | 74,4 | 100 | 95 |
| 3,86 | 71,7 | 100 | 96 | 74,5 | 100 | 95 |
| 3,89 | 71,8 | 100 | 96 | 74,6 | 100 | 96 |
| 3,92 | 72.0 | 100 | 96 | 74,7 | 100 | 96 |
| 3,95 | 72,1 | 100 | 97 | 74,9 | 100 | 96 |
| 3,97 | 72,2 | 100 | 97 | 75.0 | 100 | 96 |
| 4 | 72,3 | 100 | 97 | 75,1 | 100 | 96 |
| 4,03 | 72,4 | 100 | 97 | 75,2 | 100 | 96 |
| 4,05 | 72,5 | 100 | 97 | 75,3 | 100 | 96 |
| 4,08 | 72,7 | 100 | 97 | 75,4 | 100 | 96 |
| 4,11 | 72,8 | 100 | 97 | 75,5 | 100 | 96 |
| 4,14 | 72,9 | 100 | 97 | 75,6 | 100 | 96 |
| 4,16 | 73 | 100 | 97 | 75,7 | 100 | 97 |
| 4,19 | 73,1 | 100 | 97 | 75,8 | 100 | 97 |
| 4,22 | 73,2 | 100 | 97 | 76.0 | 100 | 97 |
| 4,24 | 73,3 | 100 | 97 | 76.0 | 100 | 97 |
| 4,27 | 73,4 | 100 | 98 | 76,2 | 100 | 97 |
| 4,3 | 73,6 | 100 | 98 | 76,3 | 100 | 97 |
| 4,32 | 73,6 | 100 | 98 | 76,4 | 100 | 97 |
| 4,35 | 73,8 | 100 | 98 | 76,5 | 100 | 97 |
| 4,38 | 73,9 | 100 | 98 | 76,6 | 100 | 97 |
| 4,41 | 74.0 | 100 | 98 | 76,7 | 100 | 97 |
| 4,43 | 74,1 | 100 | 98 | 76,8 | 100 | 97 |
| 4,46 | 74,2 | 100 | 98 | 76,9 | 100 | 97 |
| 4,49 | 74,3 | 100 | 98 | 77.0 | 100 | 98 |
| 4,51 | 74,4 | 100 | 98 | 77,1 | 100 | 98 |
| 4,54 | 74,5 | 100 | 98 | 77,2 | 100 | 98 |
| NB: RS = Raw Score; T = T-score; PR_n = Percentile Rank score general population; PR_cl = Percentile Rank score clinical sample. | | | | | | |
| ^1^Formula for population female (ID:17) for RS->TRankit: y=36.7+11764.692*(1-exp(-exp(4.776e-01*(ln(x+.0001)-ln(749730.488))))); a Weib2 function. | | | | | | |
| ^2^Formula for population female (ID:17) for RS->PR_n: y=120.1-111.264/((1+(x/14842.694)^6.779e-01)^506.529); a Weib2 function. | | | | | | |
| ^3^Formula for clinical female (ID:13) for RS->PR_cl: y=- -22.9+122.377/(1+exp(-1.283*(x-1.051))); a sigm-L5 function. | | | | | | |
| ^4^Formula for population male (ID:17) for RS->TRankit: y=40.5+12535.844*(1-exp(-exp(4.748e-01*(log(x+.0001)-log(980172.098))))); a Weib2 function. | | | | | | |
| ^5^Formula for population male (ID:17) for RS->PR_n: y=175.3-158.809/((1+(x/8986.328)^4.736e-01)^35.696);; a Weib2 function. | | | | | | |
| ^6^Formula for clinical male (ID:18) for RS->PR_cl: y=124.4-118.828/((1+(x+0.0001/2.254e-01)^2.558)^1.943e-01; a logis5 function. | | | | | | |

**Supplementary Table 10**

*Cross-Walk Table from Raw Scores to T- and PR-scores for BUT-B Positive Symptom Total*

| RS | T^1^ | PR_n^2^ | PR_cl^3^ |
| --- | --- | --- | --- |
| 0 | 37.4 | 10 | 1 |
| 1 | 41.0 | 21 | 3 |
| 2 | 42.8 | 27 | - |
| 3 | 44.2 | 32 | 7 |
| 4 | 45.4 | 36 | 9 |
| 5 | 46.5 | 39 | 12 |
| 6 | 47.5 | 43 | 14 |
| 7 | 48.5 | 46 | 16 |
| 8 | 49.4 | 48 | 19 |
| 9 | 50.2 | 51 | 21 |
| 10 | 51.0 | 53 | 24 |
| 11 | 51.8 | 56 | 26 |
| 12 | 52.5 | 58 | 29 |
| 13 | 53.2 | 60 | 31 |
| 14 | 53.9 | 62 | 34 |
| 15 | 54.5 | 64 | 37 |
| 16 | 55.2 | 66 | 40 |
| 17 | 55.8 | 68 | 42 |
| 18 | 56.4 | 70 | 45 |
| 19 | 57.0 | 72 | 48 |
| 20 | 57.6 | 73 | 51 |
| 21 | 58.2 | 75 | 54 |
| 22 | 58.7 | 77 | 57 |
| 23 | 59.3 | 78 | 60 |
| 24 | 59.8 | 80 | 63 |
| 25 | 60.4 | 81 | 66 |
| 26 | 60.9 | 83 | 69 |
| 27 | 61.4 | 84 | 72 |
| 28 | 61.9 | 86 | 75 |
| 29 | 62.4 | 87 | 78 |
| 30 | 62.9 | 88 | 81 |
| 31 | 63.4 | 90 | 84 |
| 32 | 63.8 | 91 | 87 |
| 33 | 64.3 | 92 | 89 |
| 34 | 64.8 | 94 | 92 |
| 35 | 65.2 | 95 | 94 |
| 36 | 65.7 | 96 | 97 |
| 37 | 66.1 | 97 | 99 |
| NB: RS = Raw Score; T = T-score; PR_n = Percentile Rank score general population; PR_cl = Percentile Rank score clinical sample. | | | |
| ^1^Formula for population all (ID:17) for RS->TRankit: y=37.4+22359.590*(1-exp(-exp(5.715e-01*(ln(x+.0001)-ln(4218783.215))))); a Weib2 function. | | | |
| ^2^Formula for population all (ID:17) for RS->PR_n: y=9.5+268.837*(1-exp(-exp(6.066e-01*(ln(x+.0001)-ln(171.471))))); a Weib2 function. | | | |

**Supplementary Table 10a**

*Cross-Walk Table from Raw Scores to T- and PR-scores for BUT-B Positive Symptom Total for females and males*

|  |  | female | | | male | | |
| --- | --- | --- | --- | --- | --- | --- | --- |
| RS |  | T1^1^ | PR_n1^2^ | PR_cl1^3^ | T2^4^ | PR_n2^5^ | PR_cl2^6^ |
| 0 |  | 36.8 | 9 | 1 | 40.4 | 16 | 0 |
| 1 |  | 40.5 | 19 | 2 | 44.3 | 33 | 7 |
| 2 |  | 42.3 | 25 | - | 46.0 | 39 | - |
| 3 |  | 43.7 | 30 | 6 | 47.4 | 44 | 20 |
| 4 |  | 45.0 | 34 | 8 | 48.5 | 47 | - |
| 5 |  | 46.1 | 38 | 10 | 49.5 | 50 | 30 |
| 6 |  | 47.1 | 41 | 11 | 50.4 | 53 | 35 |
| 7 |  | 48.1 | 44 | 14 | 51.3 | 56 | 39 |
| 8 |  | 49.0 | 47 | 16 | 52.1 | 58 | 43 |
| 9 |  | 49.8 | 50 | 18 | 52.8 | 60 | 46 |
| 10 |  | 50.6 | 52 | 20 | 53.5 | 62 | - |
| 11 |  | 51.4 | 55 | 23 | 54.2 | 64 | - |
| 12 |  | 52.2 | 57 | 25 | 54.8 | 66 | - |
| 13 |  | 52.9 | 59 | 28 | 55.4 | 68 | - |
| 14 |  | 53.6 | 61 | 30 | 56.0 | - | - |
| 15 |  | 54.3 | 63 | 33 | 56.6 | - | - |
| 16 |  | 54.9 | 65 | 36 | 57.2 | - | - |
| 17 |  | 55.5 | 67 | 39 | 57.7 | 74 | 67 |
| 18 |  | 56.2 | 69 | 42 | 58.2 | 76 | - |
| 19 |  | 56.8 | 71 | 45 | 58.8 | 77 | 71 |
| 20 |  | 57.4 | 73 | 49 | 59.3 | - | - |
| 21 |  | 58.0 | 75 | 52 | 59.7 | - | 74 |
| 22 |  | 58.5 | 76 | 55 | 60.2 | - | - |
| 23 |  | 59.1 | 78 | 58 | 60.7 | - | - |
| 24 |  | 59.6 | 79 | 62 | 61.2 | - | - |
| 25 |  | 60.2 | 81 | 65 | 61.6 | 85 | - |
| 26 |  | 60.7 | 82 | 68 | 62.0 | - | - |
| 27 |  | 61.2 | 84 | 72 | 62.5 | 87 | - |
| 28 |  | 61.8 | 85 | 75 | 62.9 | 88 | - |
| 29 |  | 62.3 | 87 | 78 | 63.3 | - | - |
| 30 |  | 62.8 | 88 | 81 | 63.7 | 90 | - |
| 31 |  | 63.3 | 90 | 84 | 64.1 | - | - |
| 32 |  | 63.7 | 91 | 87 | 64.5 | 92 | 87 |
| 33 |  | 64.2 | 92 | 90 | 64.9 | 93 | - |
| 34 |  | 64.7 | 93 | 92 | 65.3 | 94 | 89 |
| 35 |  | 65.2 | 95 | 95 | 65.7 | 95 | 90 |
| 36 |  | 65.6 | 96 | 97 | 66.1 | - | - |
| 37 |  | 66.1 | 97 | 99 | 66.4 | 97 | 92 |
|  | NB: RS = Raw Score; T = T-score; PR_n = Percentile Rank score general population; PR_cl = Percentile Rank score clinical sample. | | | | | | |
|  | ^1^Formula for population female (ID:17) for RS->TRankit: y=36.8+21869.378*(1-exp(-exp(5.735e-01*(ln(x+.0001)-ln(3777852.966))))); a Weib2 function. | | | | | | |
|  | ^2^Formula for population female (ID:17) for RS->PR_n: y=8.5+235.460*(1-exp(-exp(6.375e-01*(ln(x+.0001)-ln(120.206))))); a Weib2 function. | | | | | | |
|  | ^3^Formula for clinical female (ID:13) for RS->PR_cl: y=-30.5+148.589/((1+exp(-1.382e-01*(x-31.476)))^3.563e-01); a sigm-L5 function. | | | | | | |
|  | ^4^Formula for population male (ID:17) for RS->TRankit: y=40.4+14258.462*(1-exp(-exp(5.263e-01*(ln(x+.0001)-ln(5912807.838))))); a Weib2 function. | | | | | | |
|  | ^5^Formula for population male (ID:17) for RS->PR_n: y=16.4+2212.264*(1-exp(-exp(4.373e-01*(ln(x+.0001)-ln(68936.185))))); a Weib2 function. | | | | | | |
|  | ^6^Formula for clinical male (ID:20) for RS->PR_cl: y=(133.899*x)/(17.048+x); a micmen function. | | | | | | |

**Supplementary Appendix 2**

*Cut-off values for clinical significance (Reliable Change and Clinical Significant Change) according to Jacobson and colleagues (Jacobson and Truax, 1991, Jacobson et al., 1999).*

Clinical significance: cut-off values for Clinical Significant Change (CS) and Reliable Change Index (RCI) as presented in **Supplementary Table 11.**
We established cutoff values for reliable change and clinically meaningful change for raw scores on the BUT-A and BUT-B. Jacobson and colleagues developed a method for evaluating the effectiveness of a clinical treatment (Jacobson and Truax, 1991, Jacobson et al., 1999). They proposed two key indicators of clinical significance: 1. Clinically Meaningful Change (CS): This assesses whether the change is large enough to move a patient from a "clinical" or "unhealthy" range into a "healthy" range. 2. Reliable Change (RCI): This measures whether a change in a patient’s score is statistically significant, meaning it’s a genuine improvement and not just due to random chance or measurement error.

*Clinical change*

The CS cutoff value is determined by the point halfway the mean of the clinical population and the mean of the general population and is calculated as:

$CS= \frac{s_{2}*M_{1}+ s_{1}*M_{2}}{s_{1}+s_{2}}$. "*s*" is the standard deviation and "*M*" is the mean.

Patients who transition across this CS score toward a healthier state may be classified as recovered. However, a potential issue arises when a patient’s initial score is close to this cutoff. A small, random fluctuation in the score could make it appear as if they've made a significant improvement when they haven't. This apparent change is no greater than what can be expected from the inherent inaccuracy, or "noise," of the measurement tool. To address this, the Reliable Change Index (RCI) was developed. The RCI is a statistical indicator that assesses whether the change in a patient's score is large enough to be considered a genuine improvement, rather than just random variation.

*Reliable Change Index (RCI)*

RCI is calculated as: $RCI=CI*\sqrt{2{S_{E}}^{2}}$

In which: $S_{E}=\mathrm{SD}\sqrt{1-r_{xx}}$; r_xx_ is a reliability index (test-retest or, more commonly, Cronbach’s alpha; CI95 = 1.96, CI90 = 1.64, CI80 = 1.28; The chosen critical value (1.96 for a 95% confidence interval, 1.64 for 90%, and 1.28 for 80%) depends on the desired level of certainty. By combining the CS cut-off score and the RCI, treatment outcomes can be sorted into five distinct categories: recovered, merely improved, no reliable change, merely deteriorated, relapsed.

**Supplementary Table 11**

*Cut-off values for clinical significance of the BUT and its subscales*

| **BUT-A** | **a** | **SEM** | ***M_Clin_*** | ***SD_Clin_*** | ***M_pop_*** | ***SD_pop_*** | **RCI95** | **RCI90** | **RCI80** | **CSC** |
| --- | --- | --- | --- | --- | --- | --- | --- | --- | --- | --- |
| General Severity Index | 0.98 | 0.18 | 2.48 | 1.25 | 1.15 | 1.08 | 0.49 | 0.41 | 0.32 | 1.77 |
| Weight phobia | 0.92 | 0.38 | 2.94 | 1.33 | 1.34 | 1.25 | 1.04 | 0.87 | 0.68 | 2.12 |
| Body image concern | 0.94 | 0.36 | 2.68 | 1.46 | 1.26 | 1.24 | 0.99 | 0.83 | 0.65 | 1.91 |
| Avoidance | 0.88 | 0.45 | 1.95 | 1.30 | 0.84 | 1.03 | 1.25 | 1.04 | 0.82 | 1.33 |
| Compulsive self-monitoring | 0.86 | 0.52 | 2.41 | 1.39 | 1.25 | 1.12 | 1.44 | 1.21 | 0.94 | 1.77 |
| Depersonalization | 0.90 | 0.43 | 2.20 | 1.36 | 0.91 | 1.10 | 1.19 | 1.00 | 0.78 | 1.49 |
| **BUT-B** | **a** | **SEM** | ***M*** | ***SD*** | ***M*** | ***SD*** | **RCI95** | **RCI90** | **RCI80** | **CSC** |
| Positive Symptoms Total | 0.80 | 4.68 | 19.18 | 10.46 | 12.22 | 11.74 | 13.0 | 10.8 | 8.5 | 15.9 |
| Positive Symptom Distress Index | 0.96 | 0.21 | 1.45 | 1.05 | 0.79 | 0.88 | 0.58 | 0.49 | 0.38 | 1.09 |
| B1 Mouth | 0.87 | 0.41 | 1.13 | 1.13 | 0.76 | 0.97 | 1.13 | 0.94 | 0.74 | 0.93 |
| B2 Face Shape | 0.84 | 0.44 | 1.05 | 1.10 | 0.62 | 0.88 | 1.22 | 1.02 | 0.80 | 0.81 |
| B3 Thighs | 0.89 | 0.47 | 2.61 | 1.43 | 1.10 | 1.29 | 1.31 | 1.10 | 0.86 | 1.82 |
| B4 Legs | 0.82 | 0.53 | 1.40 | 1.24 | 0.69 | 0.96 | 1.46 | 1.22 | 0.95 | 1.00 |
| B5 Arms | 0.88 | 0.50 | 1.75 | 1.45 | 0.75 | 1.09 | 1.39 | 1.16 | 0.91 | 1.18 |
| B6 Moustache | 0.74 | 0.58 | 0.84 | 1.14 | 0.64 | 1.05 | 1.61 | 1.35 | 1.05 | 0.74 |
| B7 Skin | 0.70 | 0.76 | 1.58 | 1.39 | 1.13 | 1.30 | 2.11 | 1.77 | 1.38 | 1.35 |
| B8 Blushing | 0.78 | 0.53 | 1.25 | 1.12 | 0.82 | 1.00 | 1.46 | 1.22 | 0.95 | 1.02 |

M = mean, SD = standard deviation; RCI95 is Reliable Change Index at 95 % confidence interval; CSC = cut-off for Clinical Significant Change.

**References**

Cuzzolaro, M., Vetrone, G., Marano, G. & Garfinkel, P. E. 2006. The Body Uneasiness Test (BUT): Development and validation of a new body image assessment scale. *Eating and Weight Disorders - Studies on Anorexia, Bulimia and Obesity : Official Journal of the Italian Society for the Study of Eating Disorders (SISDCA),* 11**,** 1-13. doi:10.1007/BF03327738.

Jacobson, N. S., Roberts, L. J., Berns, S. B. & McGlinchey, J. B. 1999. Methods for defining and determining the clinical significance of treatment effects: description, application, and alternatives. *Journal of consulting and clinical psychology,* 67**,** 300-7.

Jacobson, N. S. & Truax, P. 1991. Clinical Significance: A Statistical Approach to Defining Meaningful Change in Psychotherapy Research. *Journal of consulting and clinical psychology,* 59**,** 12-19.
